# Exploring Phenolic Compounds as Quorum Sensing Inhibitors in Foodborne Bacteria

**DOI:** 10.3389/fmicb.2021.735931

**Published:** 2021-09-14

**Authors:** Catarina Angeli Santos, Emília Maria França Lima, Bernadette Dora Gombossy de Melo Franco, Uelinton Manoel Pinto

**Affiliations:** Department of Food and Experimental Nutrition, Food Research Center, Faculty of Pharmaceutical Sciences, University of São Paulo, São Paulo, Brazil

**Keywords:** quorum sensing, phenolics, antivirulence, antimicrobial, antibiofilm

## Abstract

The emergence of multidrug-resistant bacteria stimulates the search for new substitutes to traditional antimicrobial agents, especially molecules with antivirulence properties, such as those that interfere with quorum sensing (QS). This study aimed to evaluate the potential of phenolic compounds for QS inhibition in a QS biosensor strain (*Chromobacterium violaceum*) and three foodborne bacterial species (*Aeromonas hydrophila*, *Salmonella enterica* serovar Montevideo, and *Serratia marcescens*). Initially, an *in silico* molecular docking study was performed to select the compounds with the greatest potential for QS inhibition, using structural variants of the CviR QS regulator of *C. violaceum* as target. Curcumin, capsaicin, resveratrol, gallic acid, and phloridizin presented good affinity to at least four CviR structural variants. These phenolic compounds were tested for antimicrobial activity, inhibition of biofilm formation, and anti-QS activity. The antimicrobial activity when combined with kanamycin was also assessed. Curcumin, capsaicin, and resveratrol inhibited up to 50% of violacein production by *C. violaceum*. Biofilm formation was inhibited by resveratrol up to 80% in *A. hydrophila*, by capsaicin and curcumin up to 40% in *S.* Montevideo and by resveratrol and capsaicin up to 60% in *S. marcescens*. Curcumin completely inhibited swarming motility in *S. marcescens*. Additionally, curcumin and resveratrol increased the sensitivity of the tested bacteria to kanamycin. These results indicate that curcumin and resveratrol at concentrations as low as 6μM are potential quorum sensing inhibitors besides having antimicrobial properties at higher concentrations, encouraging applications in the food and pharmaceutical industries.

## Introduction

The microbial communication system called quorum sensing (QS) is used by a wide variety of bacteria allowing them to collectively modify their behavior in response to changes in cell density. This communication is mediated by small molecules accumulated during microbial multiplication and involves the production, secretion, and detection of extracellular signaling molecules, known as autoinducers (AI; Mukherjee and Bassler, 2019).

Gram-positive and Gram-negative bacteria exhibit different communication systems to regulate many physiological traits. In Gram-negative bacteria, signaling is usually mediated by acyl homoserine lactone (AHL) molecules, also known as autoinducer-1 (AI-1; Vanetti et al., 2020). In Gram-positive organisms, communication is mediated by autoinducer peptides which are usually secreted by ABC-type carrier proteins (Monnet et al., 2014; Lima et al., 2020).

In addition to these main QS autoinducers, the furanosyl borate diester, also known as autoinducer-2 (AI-2), is associated with both Gram-positive and Gram-negative bacteria allowing intra and interspecific communications (Chen et al., 2002). The autoinducer-3 (AI-3), a metabolite involved in pathogenesis of enterohemorrhagic *Escherichia coli* (EHEC), was recently elucidated and characterized (Kim et al., 2020). In fact, AI-3 analogs belong to the pyrazinone family, and they are derived from threonine dehydrogenase (Tdh) products and “abortive” tRNA synthetase reactions, being present in a variety of Gram-negative and Gram-positive pathogens (Kim et al., 2020). A number of other extracellular bacterial metabolites that function as signals in a range of microorganisms have also been discovered recently (Vanetti et al., 2020).

The genes and functions regulated by QS are diverse and can be classified into four groups: (1) cellular behavior, such as biofilm formation and dispersion, motility, and adhesion; (2) cell maintenance and proliferation, such as exoenzyme and siderophore synthesis, sporulation, and acid resistance; (3) horizontal gene transfer, such as conjugation in *Agrobacterium tumefaciens*; and (4) interactions with the host and other microorganisms, such as production of virulence factors, antibiotics and exo-polysaccharides, and bioluminescence, among others (Jimenez et al., 2010; Monnet et al., 2014; Grandclément et al., 2016; Lima et al., 2020).

The communication mediated by QS can be interrupted in several ways: by inhibiting the autoinducer synthesis, through enzymatic degradation of autoinducers, or by competition for binding to receptor proteins, ultimately inhibiting the target gene expression, mediated by interfering molecules called quorum sensing inhibitors (QSI; Zhang and Li, 2015; Grandclément et al., 2016).

Plants are one of the main sources of natural QSI, including medicinal plants, vegetables, and edible fruits. Some compounds derived from the secondary metabolism of plants significantly increase their ability to adapt to unfavorable environments. These metabolites, such as alkaloids, phenols, flavonoids, quinones, tannins, terpenes, and lecithins, are well-known defense mechanisms against herbivores and microorganisms (Nazir et al., 2020). Phenolic compounds are the second largest family of plant nutraceuticals, after terpenoids (Gutiérrez-Del-Rio et al., 2018). Recent studies have shown that phenolic compounds in extracts of edible foods can act as QSI (Quecán et al., 2019; Rivera et al., 2019; Santos et al., 2020).

Food spoilage causes great concern to the food industry as it leads to significant economic losses. The detection of QS signaling molecules in spoiled foods, as well as microbial interactions in fermentation of foods, has added a new dimension to the understanding of the spoilage process (Bai and Rai, 2011; Nazzaro et al., 2013; Martins et al., 2018; Almeida et al., 2020). Interference in QS communication could be an additional target to delay bacterial food spoilage since some phenotypes regulated by QS are also related to food deterioration, such as production of pectinases, lipases and proteases, and biofilm formation.

Antimicrobial resistance is one of the greatest threats to global health (WHO, 2018), and new alternatives are needed to treat infections and stop the spread of multidrug-resistant bacteria (Singh et al., 2019). The rationale for using QS inhibitors as an anti-virulence strategy is the lower selective pressure when compared to traditional antibiotics (Maura et al., 2016; Saeki et al., 2020).

This study evaluated the ability of some phenolic compounds to inhibit bacterial quorum sensing regulated phenotypes. First, we applied molecular docking with the CviR QS regulator of *C. violaceum* in order to select potential QSI, and then, we tested these compounds against several quorum sensing-regulated phenotypes.

## Materials and Methods

### *In silico* Analysis – Selection of Phenolic Compounds by Molecular Docking

Docking studies were performed according to Almeida et al. (2016, 2018). The potential anti-QS activity of 79 pre-selected compounds present in foods and plants, likely having antimicrobial and anti-QS activity (Supplementary Table 1), was evaluated, using six structural variants of the CviR QS regulator of *C. violaceum*. The crystallized structures of CviR (3QP1, 3QP2, 3QP4, 3QP5, 3QP6, and 3QP8) with different AHLs (Chen et al., 2011) were obtained from the RCSB Protein Data Bank database.^1^ The molecular structure of phenolic compounds, homoserine lactones, and furanones was obtained from the PubChem database.^2^ Molecular docking was performed using the “Dock Ligands” tool of the CLC Drug Discovery Workbench 4.0 software as described previously (Almeida et al., 2016, 2018). The five best docking scores of each compound were selected, allowing the inspection of the binding sites to the CviR protein.

### Phenolic Compound Preparation

The next steps of the study were performed with the five phenolic compounds that presented the greatest inhibitory potential in *in silico* analyses: curcumin (PubChem CID: 969516), capsaicin (PubChem CID: 1548943), gallic acid (PubChem CID: 370), resveratrol (PubChem CID: 445154), and phloridizin (PubChem CID: 4789). The compounds (Sigma-Aldrich, Brazil) were dissolved in dimethyl sulfoxide (DMSO), so that the final solvent concentration did not exceed 1% in the tests (Rivera et al., 2019). Negative control comprised DMSO at the same concentration as in the tests with phenolic compounds.

### Bacterial Strains and Growth Conditions

QS biosensor and foodborne bacterial species used in this study were *Chromobacterium violaceum* ATCC 12472, *Chromobacterium violaceum* 026 and *Serratia marcescens* MG1, cultivated at 30°C, as well as *Aeromonas hydrophila* IOC/FDA 110-36 and *Salmonella* Montevideo 163 (Monte et al., 2019), cultivated at 37°C. All strains were grown in Luria-Bertani (LB) broth (KASVI) for 18h.

### Antimicrobial Activity – Minimal Inhibitory Concentration

The antimicrobial activity of the selected phenolic compounds was evaluated by determination of the minimal inhibitory concentration (MIC), using the broth microdilution assay according to Wiegand et al. (2008), with some modifications. LB broth (100μl) containing different concentrations of each phenolic compound was added to a 96-well microtiter plate, and each well was inoculated with an overnight culture of the tested microorganism, adjusted to contain 10^5^ CFU/ml. The controls were bacterial cultures in LB broth without the compounds, LB broth with each compound in each tested concentration without bacteria (color control), and LB broth (sterility control). Growth curves were determined by measuring the optical density at 595nm (OD_595_) every 2h, during 12h on a spectrophotometer (Multiskan FC, Thermo Fisher Scientific, Waltham, Massachussetts, United States). The MIC was the lowest concentration of the compound in which there was no bacterial growth, as observed by the growth curves. All QS assays were performed in concentrations that did not interfere with bacterial growth as recommended by Defoirdt et al. (2013).

### Anti-QS Activity

#### Violacein Production

Quantification of violacein production was performed according to Santos et al. (2020), with modifications. The assay was performed in a 96-well microtiter plate containing 100μl of LB broth with each phenolic compound at sub-MIC and 10μl of inoculum containing 10^6^ CFU/ml of *C. violaceum*. The plates were incubated for 24h at 30°C at 150rpm and then dried at 50°C in a BOD incubator. Subsequently, 100μl of pure DMSO was added to each well. After 30min at room temperature, the OD_595_ was measured using a spectrophotometer (Multiskan FC, Thermo Fisher Scientific, Waltham, Massachussetts, United States). For the *C. violaceum* 026 assay, the autoinducer C6-HSL at 100μM was added to each well. LB broth without phenolic compounds was used as negative control. Results were expressed as percentages, comparing OD_595_ measurements for the tested phenolic compound and the negative control, which was considered 100%.

#### Swarming and Swimming Motility

The test was performed according to Packiavathy et al. (2014) with some modifications. Swarming motility was tested using 3ml of semi-solid LB agar 0.5% (w/v) to which the phenolic compounds were added at sub-MIC, and swimming motility was tested using 6ml of semi-solid LB agar 0.3% (w/v) with the phenolic compounds at sub-MIC. These tests were performed in tubes containing melted semi-solid LB agar mixed with the tested phenolic compound. After vortexing, the mixtures were transferred to small Petri dishes (49mm×12mm). After 10min, 2μl of a culture inoculum containing 10^8^ CFU/ml of *A. hydrophila* or *S. marcescens* was spotted at the center of the plate, followed by 24h incubation at the optimum growth temperature of the microorganism. Semi-solid agar without phenolic compound was used as control of absence of inhibition. The results of swarming and swimming motility inhibition were obtained comparing the bacterial growth diameters in the test and control plates.

#### Biofilm Formation

The analysis was performed according to Santos et al. (2020), using *S. marcescens* MG1, *A. hydrophila*, and *S*. Montevideo as target microorganisms. In a 96-well plate, 200μl of LB broth with the phenolic compound at sub-MIC was mixed with an aliquot of 20μl of an overnight culture of each tested bacterium, adjusted according to McFarland 0.5 solution (10^5^ CFU/ml). The plates were incubated for 24h at the optimal growth temperature of each bacterium. After removal of the culture medium, the plates were washed three times with 200μl of sterile saline 0.9% (w/v). The sessile cells were stained with 200μl of crystal violet 0.1% (w/v) for 30min, the dye was removed, and the wells were rinsed three times with saline solution. Each test included a negative control, correspondent to LB broth without the addition of phenolic compounds. The crystal violet retained by the adhered cells was dissolved in 200μl of 95% ethanol, and the OD_595_ nm was determined by spectrophotometry (Multiskan FC, Thermo Fisher Scientific, Waltham, Massachussetts, United States). Results were expressed as percentages, comparing OD_595_ measurements for the tested phenolic compound and the negative control, which was considered 100%.

### Sensitivity to Kanamicin

The MIC and minimum bactericidal concentration (MBC) of kanamycin (Sigma-Aldrich, Brazil) were determined using the broth microdilution method (CLSI, 2007). In a 96-well plate, 100μl of LB broth containing increasing concentrations of the antibiotic (4 to 512μg/ml) and 10μl of inoculum (10^5^ CFU/ml, according to McFarland 0.5 solution) were added to each well. Negative controls, constituted of LB broth without antibiotic, were included in each test. The plates were incubated for 24h at the optimal growth temperature of each bacterium, and the results were analyzed by visual inspection. The MIC corresponded to the concentration of the antibiotic that resulted in the absence of turbidity in the well. For confirmation of absence of viable cells, 2μl of the content of the well was inoculated on LB agar for growth visualization. The MBC value corresponded to the lowest concentration of kanamycin that prevented bacterial growth on the LB agar.

### Synergy Between Phenolic Compounds and Kanamycin

The inhibitory effect of curcumin and resveratrol in combination with kanamycin on the growth of *A. hydrophila* IOC/FDA 110-36, *S.* Montevideo 163 and *S. marcescens* MG1 was evaluated by the checkerboard method, as described by Sanhueza et al. (2017). The tests were done in 96-well plates. The wells in rows contained phenolic compounds (resveratrol ranging from 25 to 100μM and curcumin ranging from 1.5 to 50μM), and the wells in columns contained kanamycin (4 to 512μg/ml) plus LB broth, totaling 90μl in each well. Subsequently, 10μl of bacterial inoculum was added to each well (10^5^ CFU/ml, according to McFarland 0.5 solution). The last row and column were used as controls, with only one agent and increasing concentrations of the second agent. The plates were incubated for 24h at the optimal growth temperature of each bacterium, and growth curves were constructed based on OD_595_ measured every 2h using a spectrophotometer (Multiskan FC, Thermo Fisher Scientific, Waltham, Massachussetts, United States).

### Statistical Analyses

Experiments were performed in three replicates, and results were submitted to ANOVA followed by Tukey’s test using the GraphPad Prism 8.0 software. A value of *p* <0.05 was considered as statistically significant.

## Results

### Selection of Phenolic Compounds by Molecular Docking

Among the tested phenolic compounds, curcumin, capsaicin, resveratrol, and phloretin presented good molecular docking results, anchoring with good affinity to at least four of the six CviR structural variants. Based on their cost and commercial availability, they were selected for the *in vitro* studies. Gallic acid was also included due to its availability because it is a standard phenolic compound.

**Figure 1:**
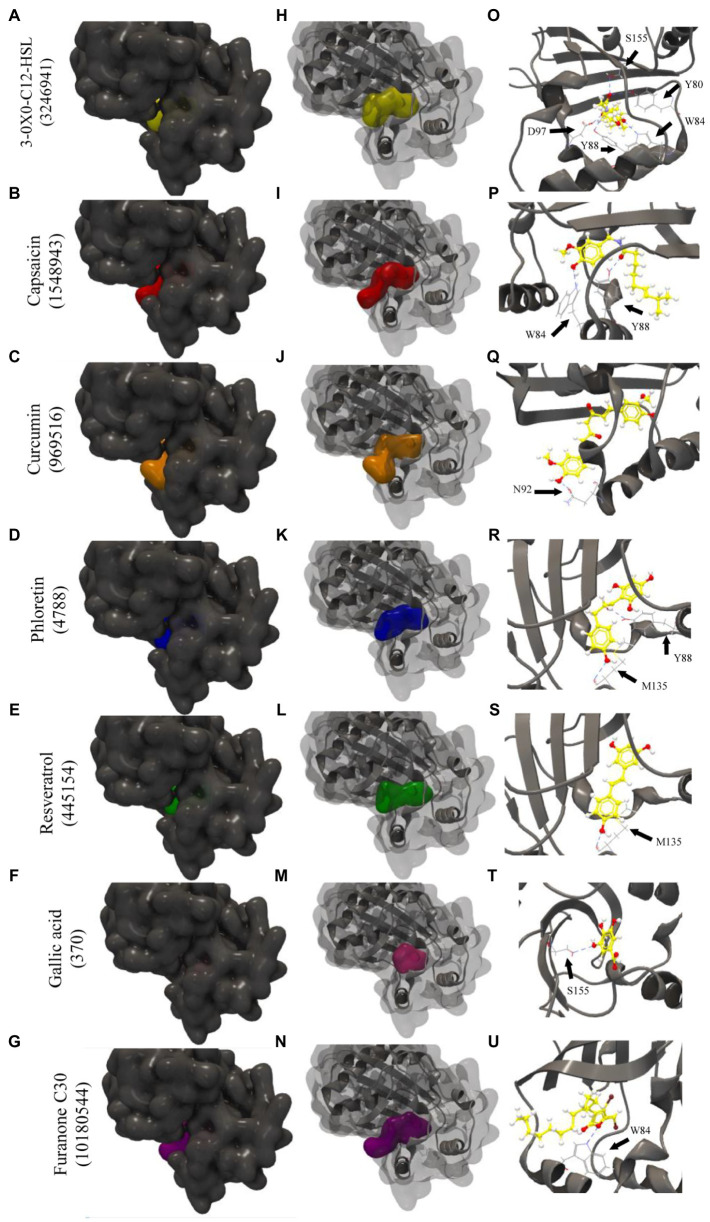
Molecular docking of the 3QP1 protein of CviR QS regulator of *Chromobacterium violaceum* with 3-oxo-C12-HSL, capsaicin, curcumin, phloretin, resveratrol, gallic acid, and Furanone C30. Surface representation **(A–G)**; surface and backbone representations **(H–N)**; and backbone representations with hydrogen bonding between amino acid residues and phenolic compounds **(O–U)**. Gray surface representation: CviR protein; yellow surface representation: 3-oxo-C12-HSL; red surface representation: capsaicin; orange surface representation: curcumin; blue surface representation: phloretin; green surface representation: resveratrol; pink surface representation: gallic acid; and purple surface representation: Furanone C30. Black arrow indicates the binding site by hydrogen bonding.

Figure 1 indicates how the tested phenolic compounds interacted with the 3QP1 protein. The interactions with the controls 3-oxo-C12-HSL (PubChem CID: 3246941) and Furanone C30 (PubChem CID: 10180544) are also shown. Ranking by the best binding affinities, 3-oxo-C12-HSL was in first place for structures 3QP1, 3QP4, and 3QP6, presenting GScores of −83.91, −85.61, and −89.6, respectively. The lower GScores (more negative) correspond to better binding affinities (Almeida et al., 2016). The phenolic compounds presented weaker binding affinity than 3-oxo-C12-HSL for all protein structures. However, the negative GScores for resveratrol (−57.30 to −66.62), capsaicin (−70.39 to −76.67), curcumin (−66.93 to −75.82), and phloretin (−63.10 to −70.19) indicated a good potential for binding to CviR QS regulator of *C. violaceum* (for the full list of GScores please see Supplementary Tables 2 and 3).

### Antibacterial Activity

#### Minimal Inhibitory Concentration

Table 1 shows that gallic acid presented a MIC of 9.4mM over the two strains of *C. violaceum* (ATCC 12472 and 026). The MIC of the other compounds was higher than the highest tested concentration. Concentrations above this limit could not be tested due to issues related to the solubility of the compounds in the assay media.

**Table 1:** Minimum inhibitory concentration (MIC) of phenolic compounds.

Target microorganism	MIC (mM)				
	Curcumin	Capsaicin	Phloridizin	Resveratrol	Gallic acid
*Aeromonas hydrophila* IOC/FDA 110-36	>0.1	>1.0	>0.9	>0.1	>9.4
*Chromobacterium violaceum* ATCC12472	>0.1	>1.0	>0.9	>0.1	9.4
*Chromobacterium violaceum* O26	>0.1	>1.0	>0.9	>0.1	9.4
*Salmonella* Montevideo 163	>0.1	>1.0	>0.9	>0.1	>9.4
*Serratia marcescens* MG1	>0.1	>1.0	>0.9	>0.1	>9.4

### Anti-QS Activity of Phenolic Compounds

#### Violacein Production

**Figure 2:**
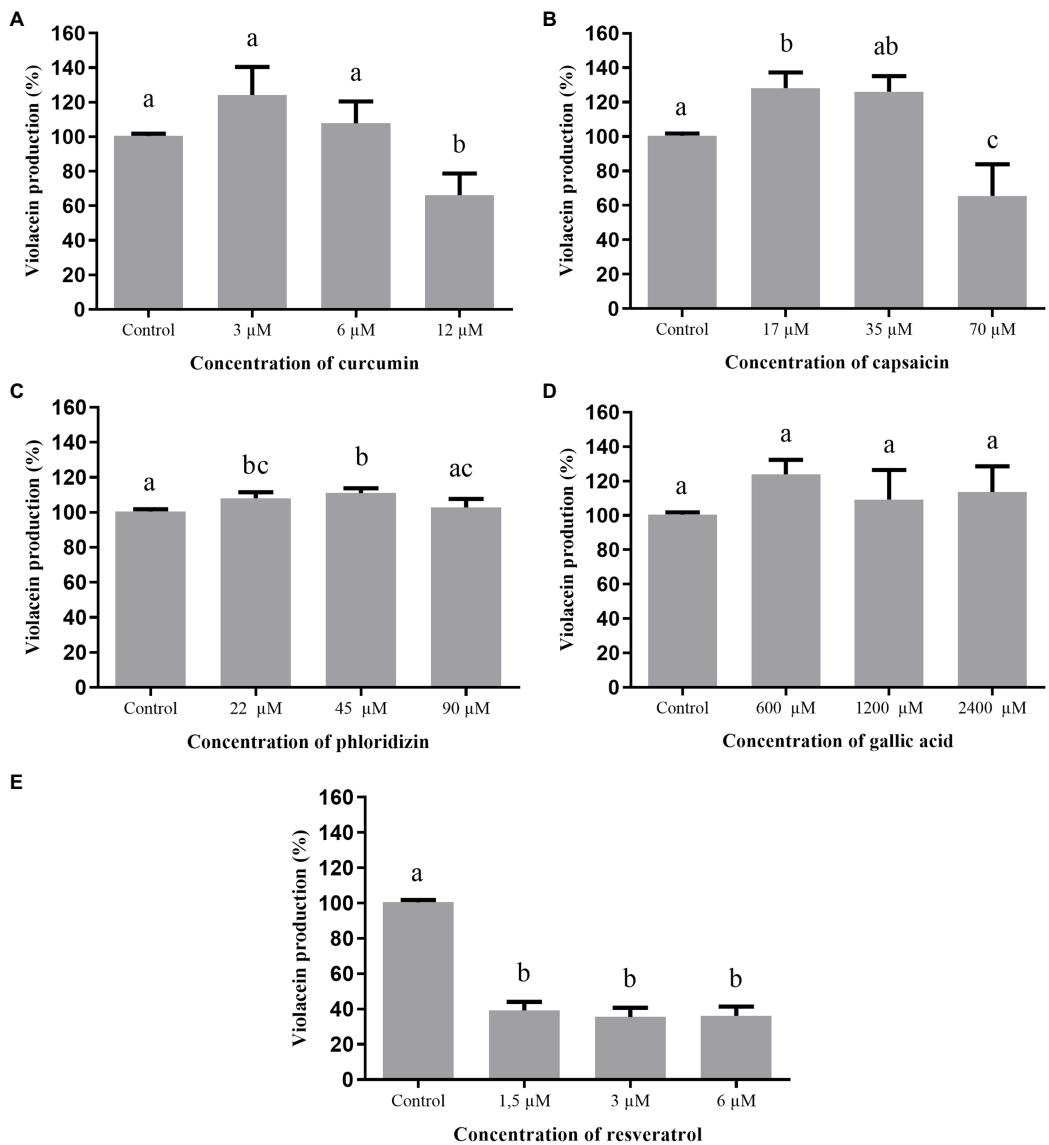
Effect of phenolic compounds on violacein production by *Chromobacterium violaceum* ATCC 12472 expressed as percentage of production, when compared to the control (100%). Curcumin **(A)**; capsaicin **(B)**; phloridizin **(C)**; gallic acid **(D)**; resveratrol **(E)**; and control=violacein production in LB broth + dimethyl sulfoxide (DMSO; 1%).

The effect of the studied phenolic compounds on violacein production by *C. violaceum* ATCC 12472 is shown in Figure 2. The inhibition of pigment production by curcumin (12μM) and capsaicin (70μM) was 40%, while resveratrol reached 60% inhibition, regardless of the tested concentration (1.5, 3.0, or 6.0μM), without affecting the growth of *C. violaceum* ATCC 12472. Gallic acid and phloridizin did not affect violacein production.

**Figure 3:**
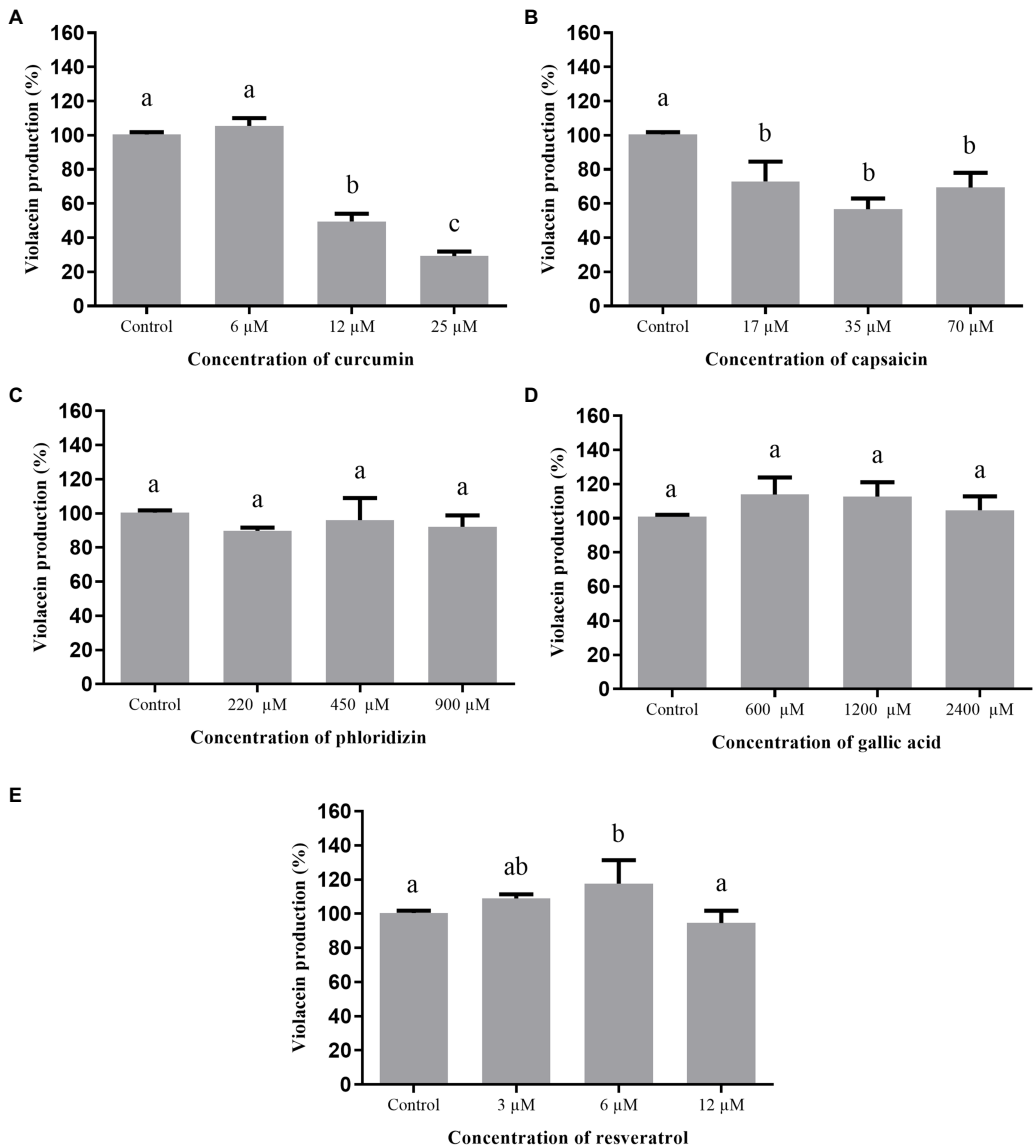
Effect of phenolic compounds on violacein production by *Chromobacterium violaceum* 026 in the presence of C6-HSL, expressed as percentage of production, when compared to the control (100%). curcumin **(A)**; capsaicin **(B)**; phloridizin **(C)**; gallic acid **(D)**; resveratrol **(E)**; and control=violacein production in LB broth + DMSO (1%).

Figure 3 shows the effect of the phenolic compounds on violacein production by *C. violaceum* 026 in the presence of C6-HSL. *C. violaceum* 026 is not capable of producing the autoinducer C6-HSL due to a mutation in the *cviI* gene but maintains the ability to receive exogenous short-chain HSLs (4 to 6 carbons) as the *cviR* gene remains functional, resulting in production of the violacein pigment (Zhang et al., 2016). Among the evaluated phenolic compounds, only curcumin and capsaicin inhibited the production of violacein by this strain. The inhibition presented by curcumin was concentration-dependent, in which the highest tested concentration (25μM) inhibited 75% of pigment production.

As phloridizin and gallic acid did not affect violacein production, indicating that they did not present any anti-QS activity, they were excluded from the tests for motility and biofilm formation.

#### Swarming and Swimming Motility

Table 2 shows the effect of phenolic compounds on the swarming motility of *A. hydrophila* and *S. marcescens*. *Salmonella* Montevideo was unable to perform swarming motility in our study. Capsaicin and resveratrol did not inhibit the swarming motility of *A. hydrophila* (*p* >0.05) at the evaluated concentrations, while curcumin induced the motility in this organism (*p* <0.05). Conversely, curcumin effectively inhibited the swarming motility of *S. marcescens*, indicating the anti-QS potential of curcumin in this organism. In contrast, capsaicin and resveratrol did not inhibit this phenotype (*p* >0.05; Supplementary Figure 1S).

**Table 2:** Effect of capsaicin, curcumin, and resveratrol on swarming motility of *Aeromonas hydrophila* IOC/FDA 110-36 and *Serratia marcescens* MG1.

Phenolic compound	Radius of swarming zone after 12 h (mm)			
	Tested concentration	*Aeromonas hydrophila*	Tested Concentration	*Serratia marcescens*
Capsaicin	0μM	12.0 ± 0.0^a^	0μM	20.0 ± 0.0^a^
	65μM	12.2 ± 0.3^a^	65μM	30.0 ± 1.4^b^
	130μM	9.5 ± 2.1^a^	130μM	31.5 ± 0.7^b^
	260μM	8.7 ± 1.0^a^	260μM	32.5 ± 0.7^b^
Curcumin	0μM	12.5 ± 0.7^ab^	0μM	49.0 ± 0.0^a^
	3μM	15.2 ± 1.0^abc^	12.5μM	5.5 ± 0.7^b^
	6μM	16.5 ± 0.0^bc^	25μM	5.0 ± 0.0^b^
	12μM	18.7 ± 3.1^c^	50μM	5.0 ± 1.4^b^
Resveratrol	0μM	13.5 ± 2.1^a^	0μM	22.5 ± 2.1^ab^
	25μM	14.0 ± 0.0^a^	25μM	31.0 ± 1.4^b^
	50μM	13.7 ± 0.3^a^	50μM	27.0 ± 2.8^b^
	100μM	14.0 ± 0.0^a^	100μM	21.0 ± 0.0^ab^

*Results expressed as mean±standard deviation*.

*Means followed by different superscript letters (a,b,c) in the same column differ among themselves by Tukey’s test (p<0.05)*.

Regarding swimming motility, none of the tested phenolic compounds inhibited this phenotype in *A. hydrophila* and *S. marcescens* (*p* >0.05; Supplementary Figure 2S).

#### Biofilm Formation

**Figure 4:**
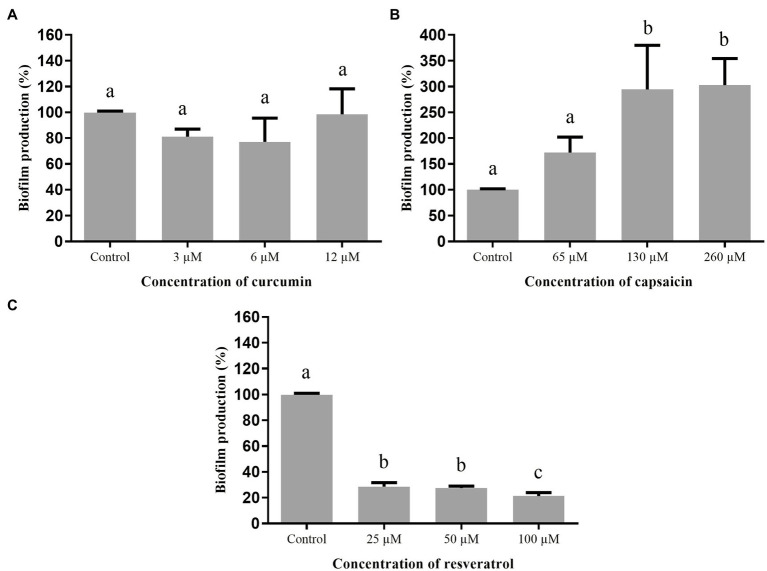
Effect of phenolic compounds on biofilm formation by *Aeromonas hydrophila* IOC/FDA 110-36, expressed as percentage of production, when compared to the control (100%). curcumin **(A)**; capsaicin **(B)**; resveratrol **(C)**; and control=biofilm formation in LB broth + DMSO (1%). Means followed by different letters differ among themselves by Tukey’s test (*p* <0.05).

The effect of curcumin, capsaicin, and resveratrol on biofilm formation by *A. hydrophila* is shown in Figure 4. Curcumin (Figure 4A) and capsaicin (Figure 4B) did not inhibit this phenotype. In fact, capsaicin induced the biofilm formation in a concentration-dependent manner. In contrast, resveratrol (Figure 4C) significantly reduced the biofilm formation up to 80% at 100μM.

**Figure 5:**
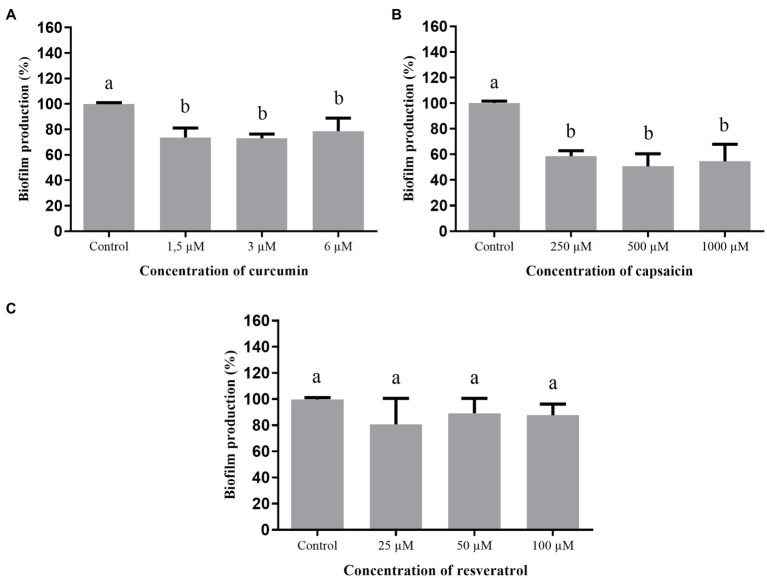
Effect of phenolic compounds on biofilm formation by *Salmonella* Montevideo 163 expressed as percentage of production, when compared to the control (100%). curcumin **(A)**; capsaicin **(B)**; resveratrol **(C)**; and control=biofilm formation in LB broth + DMSO (1%). Means followed by different letters differ among themselves by Tukey’s test (*p* <0.05).

Figure 5 shows the effect of curcumin, capsaicin, and resveratrol on biofilm formation by *S.* Montevideo. Inhibitory activity of curcumin (Figure 5A) and capsaicin (Figure 5B) was similar; i.e., both were able to inhibit biofilm formation up to 27% when compared to the control (*p* <0.05). In contrast, resveratrol (Figure 5C) did not inhibit biofilm formation at the tested concentrations (*p* >0.05).

**Figure 6:**
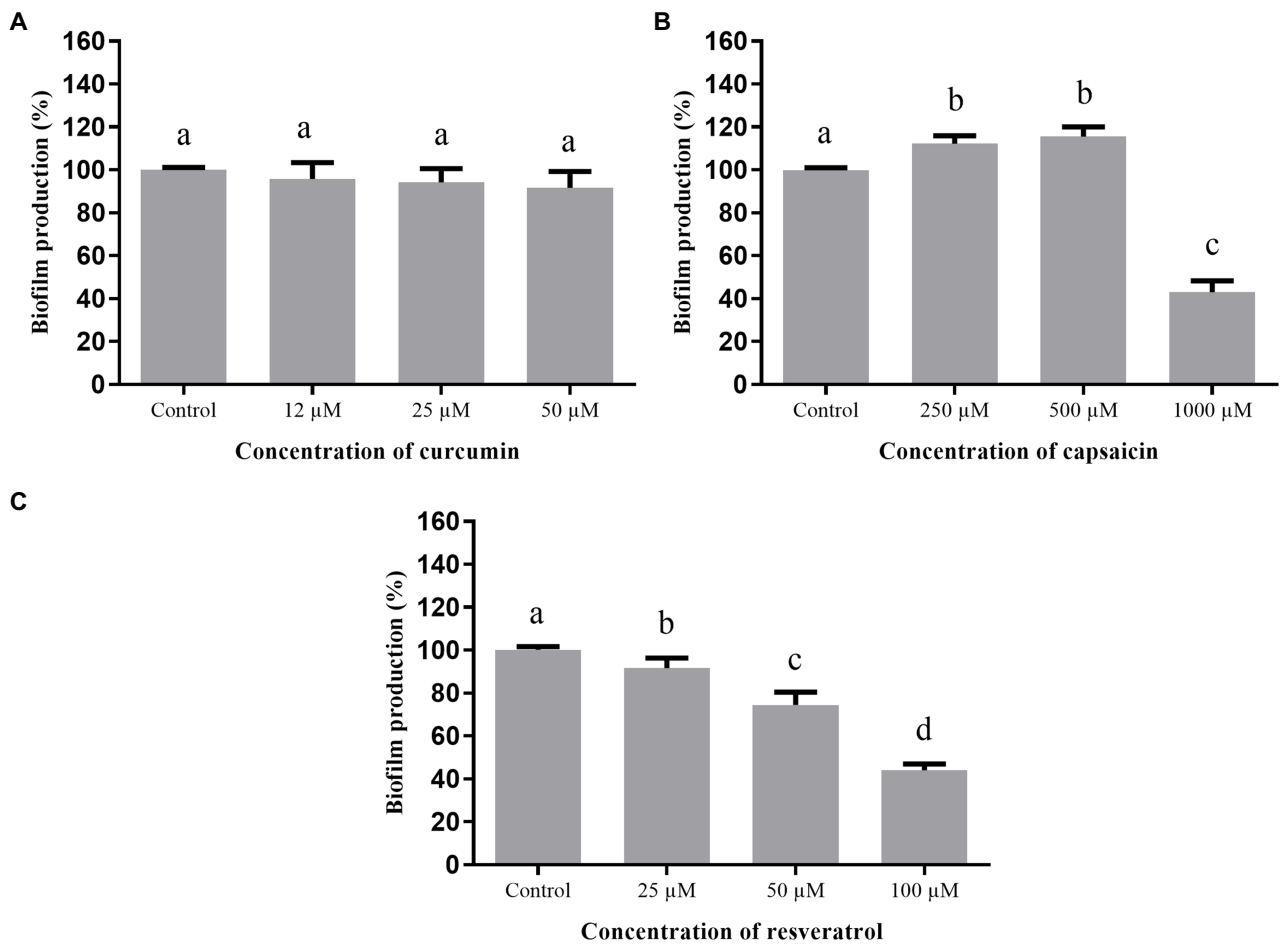
Effect of phenolic compounds on biofilm formation by *Serratia marcescens* MG1 expressed as percentage of production, when compared to the control (100%). Curcumin **(A)**; capsaicin **(B)**; resveratrol **(C)**; and control=biofilm formation in LB broth + DMSO (1%). Means followed by different letters differ among themselves by Tukey’s test (*p* <0.05).

When the phenolic compounds were tested for inhibition of biofilm formation by *S. marcescens* (Figure 6), curcumin (Figure 6A) was ineffective (*p* >0.05). Capsaicin (Figure 6B) and resveratrol (Figure 6C) were active at the highest tested concentrations; i.e., 1,000μM capsaicin and 100μM resveratrol were able to inhibit biofilm formation up to 43%.

### Effect of Phenolic Compounds (Curcumin and Resveratrol) Combined With Kanamycin on Growth

**Figure 7:**
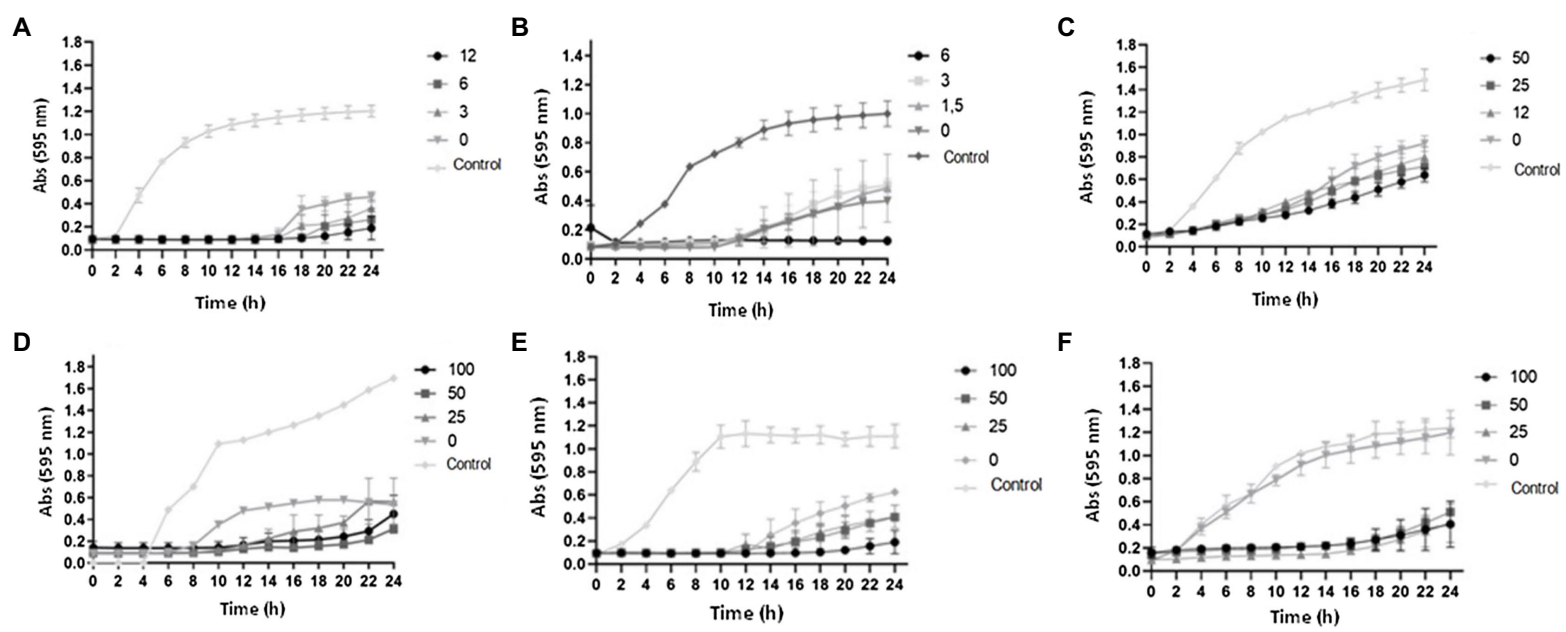
Growth curves of bacteria treated with phenolic compounds and kanamycin. **(A)** *A. hydrophila* IOC/FDA 110-36 treated with curcumin (0, 3, 6, and 12μM) and kanamycin (256μg/ml). **(B)** *Salmonella* Montevideo 163 treated with curcumin (0, 1.5, 3, and 6μM) and kanamycin (32μg/ml). **(C)** *Serratia marcescens* MG1 treated with curcumin (0, 12, 25, and 50μM) and kanamycin (32μg/ml). **(D)** *A. hydrophila* IOC/FDA 110-36 treated with resveratrol (0, 25, 50, and 100μM) and kanamycin (128μg/ml). **(E)** *Salmonella* Montevideo treated with resveratrol (0, 25, 50, and 100μM) and kanamycin (32μg/ml). **(F)** *Serratia marcescens* treated with resveratrol (0, 25, 50, and 100μM) and kanamycin (4μg/ml). Control=bacterial growth in LB broth + DMSO (1%).

Figure 7 presents the growth curves of *A. hydrophila*, *S.* Montevideo, and *S. marcescens* in LB broth containing curcumin or resveratrol in combination with kanamycin, at varied concentrations. The curves clearly indicate that the two phenolic compounds delayed the growth of all tested bacteria when combined with sub-inhibitory concentrations of the antibiotic, in a concentration-dependent pattern.

For *A. hydrophila*, 256μg/ml of kanamycin held the bacterial growth for at least 14h. Growth in the presence of curcumin (3–12μM) was slowed for longer periods of time (Figure 7A). Lower tested concentrations of kanamycin (4–128μg/ml) did not affect the growth of this bacterium (Supplementary Figure 3S). When the concentration of kanamycin was increased to 512μg/ml, there was no bacterial growth, representing the MBC for this antibiotic.

In the tests with *S.* Montevideo, 6μM of curcumin and 32μg/ml of kanamycin completely inhibited the bacterial growth (Figure 7B). Lower concentrations of curcumin (0–3μM) or kanamycin (4–16μg/ml) had no pronounced effect on growth (Supplementary Figure 4S). Higher concentrations of kanamycin (64 to 512μg/ml) caused the complete inhibition of bacterial growth (Supplementary Figure 4S).

For *S. marcescens*, the combination of curcumin with kanamycin in inhibiting growth was less effective than for *A. hydrophila* and *S.* Montevideo (Figure 7C). Evidence of inhibition was observed only when 32μg/ml of kanamycin was combined with curcumin, being slightly more effective at 50μM of the later. Lower concentrations of kanamycin (4 to 16μg/ml) and curcumin (0–50μM) resulted in no reduction in the microbial growth, while higher concentrations of kanamycin (64 to 512μg/ml) resulted in stronger growth inhibition that was not affected by the presence of curcumin (Supplementary Figure 5S).

Results of the tests with resveratrol (0–100μM) combined with kanamycin (4–512μg/ml) are shown in Figure 7D–F. The growth curves of *A. hydrophila* (Figure 7D), *S*. Montevideo (Figure 7E), and *S. marcescens* (Figure 7E) indicate that the effects of these combinations were similar to those observed for the curcumin-kanamycin combinations.

The curves from assays with kanamycin (4 to 64μg/ml) and resveratrol (0–100μM) show partial inhibition of growth of *A. hydrophila* from 8 to 10h, compared with the curve from assays using only kanamycin and the control (Supplementary Figure 6S). Kanamycin at 128μg/ml combined with resveratrol presented a growth delay, in which the bacterial growth started at 14h of incubation, while with the treatment containing only kanamycin the growth started at 8h of incubation (Figure 7D). The results show that resveratrol made *A. hydrophila* more sensitive to kanamycin at the studied concentrations (Supplementary Figure 6S).

The lowest concentrations of kanamycin (4 and 8μg/ml) did not inhibit the growth of *S*. Montevideo. In the concentration of 32μg/ml of kanamycin, there was growth retardation in all concentrations of resveratrol evaluated, especially at 100μM, with a delay of 6h compared to the other treatments (Figure 7E). In the highest concentrations of antibiotic, there was no microbial growth; thus, 64μg/ml is the MBC of this microorganism (Supplementary Figure 7S).

In the test with *S. marcescens*, at concentrations from 4 to 16μg/ml of kanamycin, growth in the presence of resveratrol was strongly inhibited. The highest concentration of resveratrol (100μM) reduced the MIC 3-fold when compared to the curve containing only kanamycin (Figure 7F). At concentrations of 32 to 128μg/ml of kanamycin, resveratrol completely inhibited growth. It was observed that resveratrol made the microorganism more sensitive to the antibiotic. Finally, complete inhibition of growth was observed in the presence of 256 and 512μg/ml kanamycin alone (Supplementary Figure 8S).

## Discussion

Phenolic compounds are known for their antimicrobial effects as a consequence of structural or functional damages to the bacterial cell membrane (Silva et al., 2018). Besides, phenolic acids can affect the physicochemical properties of the bacterial cell surface, especially hydrophobicity, in addition to altering electron receptors and polar and non-polar components of bacteria (Borges et al., 2013). Furthermore, phytochemicals, such as phenolic compounds, may compete with AHL signaling molecules for binding to QS receptors (Nazzaro et al., 2013; Almeida et al., 2018; Aswathanarayan and Vittal, 2018; Quecán et al., 2018; Santos et al., 2020), suggesting possible interactions of these compounds with LuxR-type proteins, like the CviR QS regulator of *Chromobacterium violaceum* (Figure 1).

Curcumin, one of the selected phenolic compounds for the *in vitro* studies, is the main polyphenol in the rhizome of *Curcuma longa*. Beyond its numerous pharmaceutical properties, such as antitumor, anti-HIV-1, and antibacterial activity, this compound also presents anti-QS activity, inhibiting several phenotypes in *Pseudomonas aeruginosa* PAO1, *Escherichia coli*, *Serratia marcescens*, and *Proteus mirabilis* (Ding et al., 2017). Resveratrol is also active against various pathogens and presents anti-QS activity, being capable of inhibiting biofilm formation and dispersing established biofilms (Duarte et al., 2015). Gallic acid is a phenolic acid present in numerous foods, and also an antimicrobial agent, affecting bacterial cell membranes and causing irreversible changes in permeability, rupture, and pore formation (Sorrentino et al., 2018). Capsaicin and luteolin are polyphenols abundant in several pepper species, especially those of the *Capsicum* genus, and have the ability to inhibit microbial growth, in addition to antioxidant, anti-inflammatory, cardioprotective, neuroprotective, and anticancer activity (Basith et al., 2016; Rivera et al., 2019).

The antimicrobial activity of phloridizin in the tested concentrations against the target microorganisms was lower when compared to the other phenolic compounds. Barreca et al. (2014) evaluated the antimicrobial activity of phloridizin and phloretin, its aglycone form, against several Gram-positive and Gram-negative bacteria, and observed that the presence of glucose in the basic chalcone structure caused a reduction in antimicrobial activity. Furthermore, they also observed that phloretin is particularly active against Gram-positive bacteria. The low activity against the tested Gram-negative microorganisms observed in this study corroborate the findings of Barreca et al. (2014). Interestingly, Quecán et al. (2019) also observed that glycosylation of quercetin reduces its antimicrobial and anti-QS effect.

Regarding the determination of the MIC of phenolic compounds, it is crucial to perform QS assays in concentrations that do not interfere with bacterial growth, since this would affect quantification of QS regulated phenotypes due to cell density differences (Defoirdt et al., 2013; Maura et al., 2016). Bali et al. (2019) evaluated the MIC of curcumin (1,500μg/ml, equivalent at 4.1mM) and gallic acid (>1,500μg/ml, equivalent to >8.8mM) in *C. violaceum* ATCC 12472, corroborating the results of the present work (Table 1). Packiavathy et al. (2014) also evaluated anti-QS activity of curcumin and reported an MIC of 384μg/ml, equivalent to 1.04mM, for *C. violaceum* 026, a value higher than that tested in the present study (0.1mM). Rivera et al. (2019) studied compounds present in *Capsicum frutescens* pepper extract and found that MIC of capsaicin was higher than 100μg/ml (equivalent to 0.34mM). The growth of *C. violaceum* ATCC 12472 and 026 was partially inhibited by the compound at 0.1mM, which is similar to the results found in the present work.

Curcumin inhibited violacein production by *C. violaceum* ATCC 12472 (Figure 2), as also observed by Bali et al. (2019). However, those authors evaluated higher concentrations (2mM, 1.02mM, and 0.51mM) than those used in the present work (0.012mM, 0.006mM and 0.003mM), indicating that even at lower concentrations, curcumin consistently inhibits this QS regulated phenotype. It should be noted that in concentrations higher than 0.050mM, a partial inhibition of microbial growth was observed in our study (results not shown), which should affect violacein production in a QS-independent manner.

In the study of Rivera et al. (2019), capsaicin did not inhibit violacein production when the concentration was similar to the one used in this study. However, those authors did not test the concentration of 70μM, which was the one with best anti-QS activity detected here. When working with resveratrol, Duarte et al. (2015) found positive results for violacein inhibition, corroborating the results of this work and the potential of this compound. They further suggested that the anti-QS activity of resveratrol is due to its ability to mimic QS signals and disrupt bacterial communication (Duarte et al., 2015). In the present study, phloridizin and gallic acid did not show anti-QS activity in *C. violaceum* ATCC 12472. Borges et al. (2014) and Bali et al. (2019) also reported that gallic acid did not exhibit anti-QS activity in the biosensor strain.

Tests performed with only wild-type *C. violaceum* strain are not enough to detect the QS inhibitory mechanism, since the inhibition of violacein production may be due to reduced production of the autoinducer (inhibition of AHL synthesis by CviI) or by interference with the AHL-dependent transcriptional activator, the CviR protein (Rivera et al., 2019). Thus, besides *C. violaceum* ATCC 12472, complementary assays performed with additional strains, such as *C. violaceum* 026, a mutant unable to produce AHLs but still capable to respond to exogenous AHLs, can be informative (Rivera et al., 2019). If the tested compounds interfere with C6-HSL detection by CviR, a reduced violacein production by *C. violaceum* 026 can be expected (LaSarre and Federle, 2013). Thus, curcumin and capsaicin likely present anti-QS activity *via* AHL detection by CviR since these compounds reduced violacein production in *C. violaceum* 026 (Figure 3). Brackman et al. (2009) and Packiavathy et al. (2014) also observed inhibition of violacein production by curcumin in *C. violaceum* 026, even though they have used higher concentrations than those used in this study.

Swarming motility is an organized microbial movement on surfaces, dependent on extensive flagellation, cell–cell contact, and QS. This type of motility is associated with virulence and antibiotic resistance of various microorganisms, and is considered a favorable adaptation to the challenges that arise in dynamic environments, contributing to biofilm formation and infection (Rütschlin and Böttcher, 2019; Carette et al., 2020). Interference with this phenotype is an important alternative to reduce or prevent biofilm-based infections. In this study, curcumin effectively inhibited swarming motility (Table 2 and Supplementary Figure S1), which corroborates results reported by Packiavathy et al. (2014), who observed the inhibition of swarming motility of *S. marcescens*, *E. coli*, *P. aeruginosa*, and *P. mirabilis* by curcumin at 100μg/ml (135μM). One explanation for the observed inhibition is the ability of curcumin in reducing the production of extracellular polysaccharides by some pathogens, negatively affecting their motility (Zheng et al., 2020).

Swimming-type inhibition by curcumin liposomes was also observed for *A. hydrophila* and *Serratia grimesii* (Ding et al., 2018) and for *S. marcescens*, *E. coli*, *P. aeruginosa*, and *P. mirabilis* (Packiavathy et al., 2014). Other studies also observed motility inhibition by phenolic compounds, like proanthocyanidins and tannins that completely inhibited swarming, but did not block swimming (O’May and Tufenkji, 2011), and trans-resveratrol, that had greater activity on swarming than on swimming motility, indicating that these movement phenotypes have different activation mechanisms.

Regarding the inhibition of biofilm formation by *A. hydrophila* (Figure 4), only resveratrol showed a significant effect. Similar results were found in other studies using different bacteria: *L. monocytogenes* (Vasquez-Armenta et al., 2018); *Campylobacter* spp. and *Arcobacter butzleri* (Duarte et al., 2015); methicillin-resistant *S. aureus* (MRSA; Qin et al., 2014); and *E. coli* O157: H7 (Lee et al., 2013), evidencing the broad inhibitory effect of resveratrol.

Mangoudehi et al. (2020) evaluated curcumin at different concentrations against biofilm formation of *A. hydrophila* ATCC 7966 and other three strains isolated from fish (silver carps) and found 67% reduction of biofilm formation at 43.4μM. These results differ from those observed in the present study (Figure 4A), where the maximum concentration was 12μM, indicating that biofilm inhibition is concentration-dependent. On the other hand, curcumin inhibited the biofilm formation by *S.* Montevideo (Figure 5). The efficacy of curcumin against various infectious organisms results from multiple mechanisms, including its ability to disrupt bacterial membranes, inhibit replication, and alter gene expression (Vaughn et al., 2017). Packiavathy et al. (2014) observed a visible reduction in microcolonies, disruption of biofilm architecture, and reduced biofilm biomass of *P. aeruginosa*, *S. marcescens*, *E. coli*, and *P. mirabilis* treated with curcumin.

Biofilm formation by *S.* Montevideo was also inhibited by capsaicin (Figure 5). This compound is used as a food additive and flavoring, and has important pharmacological actions, such as bacteriostatic activity against Gram-negative bacteria and anti-inflammatory effects (Zhou et al., 2014). Some studies have shown that capsaicin inhibits adhesion, growth, and biofilm formation of Gram-negative bacteria, such as *Pseudomonas putida*, *Vibrio matriegens*, *Vibrio parahaemolyticus* (Xu et al., 2005), and *Porphyromonas gingivalis* (Zhou et al., 2014).

Regarding the inhibition of biofilm formation of *S. marcescens* (Figure 6) by capsaicin at 1,000μM and resveratrol at 25μM or higher, interesting effects were observed. The capability of trans-resveratrol to inhibit biofilm formation by enterohemorrhagic *E. coli* O157:H7 ATCC43895 (Lee et al., 2013), *P. aeruginosa* PAO1 and PA14 (Cho et al., 2013), and *S. aureus* (Morán et al., 2014) has already been described. Thus, resveratrol can be considered a potential anti-biofilm compound for several biofilm-forming microorganisms and its use should be further investigated on food-contacting materials.

Antimicrobial resistance is a serious concern in clinical practice. One strategy that is useful in combating multidrug-resistant bacteria is the coadministration of antibiotics with adjuvants that modify bacterial resistance. Several medicines of great utility have been approved using this concept, such as the use of amoxicillin with beta-lactamase inhibitors. Other studies also show the effective combination of antibiotics with efflux-pump inhibitors (Ayaz et al., 2019). As biofilm formation is associated with reduced sensitivity to antibiotics, and it is known that QS regulates the expression of some genes associated with this phenotype, QS interference can be explored as an alternative to reduce bacterial virulence and increase the efficacy of antibiotic treatment (Mion et al., 2019). This study showed that curcumin at 6μM reduced the MIC of kanamycin by half in *S.* Montevideo (Figure 7B). These results corroborate those of Packiavathy et al. (2014) who observed increased sensitivity of *S. marcencens* to different antibiotics when combined with curcumin. Mun et al. (2013) and Kali et al. (2016) also observed a decrease in the MIC of several antibiotics against MRSA and other bacteria, when applied in combination with curcumin. These studies demonstrate that this phenolic compound can increase the sensitivity of several pathogens to antibioticos and is a potential agent to be coadministered with these drugs.

*Serratia marcescens* may be resistant to several antibiotics, including beta-lactam, aminoglycosides, and fluoroquinolones, hindering the treatment against infections caused by this bacterium (Yang et al., 2012). Like other Enterobacteriaceae, the production of beta-lactamase enzyme which inactivate beta-lactam antibiotics is the most common resistance mechanism in this micro-organism (Yang et al., 2012). The increased sensitivity of *S. marcescens* to colistin when combined with resveratrol reported by Cannatelli et al. (2018), the potentiation of the effects of aminoglycosides against biofilms of *P. aeruginosa* PAO1 (Zhou et al., 2018), and the increased sensitivity of multidrug resistant *Klebsiella pneumoniae* and *E. coli* (Liu et al., 2020b) and *S. aureus* (Liu et al., 2020a) deserve to be better explored as effective therapeutic alternatives.

The combination of bioactive compounds with antimicrobials seems to be a promising approach to improve the efficacy of these drugs. However, there are several challenges to be faced before translating these *in vitro* findings into real-life applications. Some of the challenges we envision are related to interactions and metabolization of these compounds in the human body, which could hinder their antimicrobial and anti-quorum sensing activities *in vivo*. Additionally, as pointed out by Vipin et al. (2020) and observed in the present work, strain variation in response to bioactive compounds is another challenge in treating infections.

## Conclusion

Phenolic compounds have potential to inhibit quorum sensing in foodborne bacteria. *In silico* analyses showed that gallic acid, capsaicin, curcumin, phloridizin, and resveratrol could interact with CviR QS receptor of *C. violaceum*. Curcumin and resveratrol presented better activity against violacein production, swarming motility, biofilm formation, and concomitant use with an antibiotic for increased microbial sensitivity. The present study and several others from the literature indicate that curcumin and resveratrol are potential QSI and have antimicrobial properties that encourage studies that could translate these findings to applications in the food and pharmaceutical industries. In order to characterize the specificity of quorum sensing inhibition, biochemical and genetic studies should be performed with these compounds and model organisms.

## Data Availability Statement

The raw data supporting the conclusions of this article will be made available by the authors, without undue reservation.

## Author Contributions

All authors have made direct and intellectual contribution to the work and approved it for publication.

## Funding

We thank São Paulo Research Foundation (FAPESP 2013/07914-8) and National Council for Scientific and Technological Development (CNPq-Brazil-grant #422242/2018-7 and CNPQ 430439/2018-0) for financial support. We acknowledge QIAGEN Company for the license to use the CLC Drug Discovery Workbench 4.0 software. CS and EL acknowledge CNPq-Brazil and the Coordination for the Improvement of Higher Education Personnel (CAPES-Brazil) for scholarships.

## Conflict of Interest

The authors declare that the research was conducted in the absence of any commercial or financial relationships that could be construed as a potential conflict of interest.

## Publisher’s Note

All claims expressed in this article are solely those of the authors and do not necessarily represent those of their affiliated organizations, or those of the publisher, the editors and the reviewers. Any product that may be evaluated in this article, or claim that may be made by its manufacturer, is not guaranteed or endorsed by the publisher.

## Footnotes

^1^ http://www.rcsb.org

^2^ http://pubchem.ncbi.nlm.nih.gov
