# Supplementary material for: Exploring Phenolic Compounds as Quorum Sensing Inhibitors in Foodborne Bacteria
Source: Front Microbiol. 2021 Sep 14;12:735931. doi: 10.3389/fmicb.2021.735931 (PMC8477669; doi:10.3389/fmicb.2021.735931)
Supplement: Supplementary file 1 [file Data_Sheet_1.docx]

**Supplementary Table 1:** Bioactive compounds selected from the literature

| **Bioactive compound** | **PubChem CID** | **Beneficial activity** | **Doi** |
| --- | --- | --- | --- |
| Hidroquinone | 785 | Antibacterial activity | 10.1089 / mdr.2018.0149 |
| 4-hydroxybenzoic acid | [135](https://pubchem.ncbi.nlm.nih.gov/compound/135) | Antibacterial and antifungal activity | 10.5539 / jas.v1n2p15 |
| Amentoflavone | 5281600 | Antibacterial activity | 10.1016/j.foodchem.2018.07.159 |
| Apigenin | 5280443 | Antibacterial and antibiofilm, inhibition of AI-1 and AI-2 | 10.1111/j.1365-2672.2010.04677.x |
| Arbutin | [440936](https://pubchem.ncbi.nlm.nih.gov/compound/440936#section=Top) | Antibacterial and antitumor activity | 10.1016/j.phytol.2015.05.015 |
| Baicalein | [5281605](https://pubchem.ncbi.nlm.nih.gov/compound/5281605) | Antibacterial and antibiofilm activity, bactericidal | 10.1371/journal.pone.0153468 |
| Bilobetin | 5315459 | Antifungal activity | 10.1515 / znc-2003-1-212 |
| Bisdemethoxycurcumin | 5315472 | Anticancer activity | [10.1021/om500317b](https://doi.org/10.1021/om500317b) |
| BR-xanthone | [13964005](https://pubchem.ncbi.nlm.nih.gov/compound/13964005#section=Top) | Antifungal activity | 10.1021/np970165u |
| Caffeic acid | 689043 | Antimicrobial activity and inhibition of violacein | 10.1080/08927014.2013.852542 |
| Capsaicin | 1548943 | Antivirulence and antibacterial activity, | 10.3389 / fmicb.2015.01281 |
| Chlorogenic acid | [1794427](https://pubchem.ncbi.nlm.nih.gov/compound/1794427) | Inhibition of violacein production | 10.1016/j.foodcont.2011.09.006 |
| Cianidanol | 9064 | Antibacterial activity | 10.2101/Ajid.12v1S.8 |
| Curcumin | [969516](https://pubchem.ncbi.nlm.nih.gov/compound/969516) | Antibacterial activity, anticancer and anti-inflammatory | 10.1016 / j.jgar.2017.03.006 |
| Cyanidin | [128861](https://pubchem.ncbi.nlm.nih.gov/compound/128861) | Antibiofilm activity, inhibition of violacein and EPS production | 10.1007 / s13197-015-2031-9 |
| Cyanidin-3-glucoside | [441667](https://pubchem.ncbi.nlm.nih.gov/compound/441667) | Anti-inflammatory activity | 10.1007/s10068-014-0279-x |
| Daidzein | 5281708 | Inhibition of violacein production | 10.1016/j.foodcont.2011.09.006 |
| Delphinidin-3-glucoside | [102515359](https://pubchem.ncbi.nlm.nih.gov/compound/102515359) | Antioxidant and antibacterial activity | [10.3390/molecules20045698](https://dx.doi.org/10.3390%2Fmolecules20045698) |
| Delphinidin | 128853 | Anti-inflammatory and antibiofilm activity | 10.1016/B978-0-12-805417-8.00023-8 |
| Demethoxycurcumin | [5469424](https://pubchem.ncbi.nlm.nih.gov/compound/5469424#section=Top) | Anti-inflammatory and anti infectious activity | 10.1002/jcp.28626 |
| Dihydrocapsaicin | 107982 | Antibacterial activity | 10.1007 / s11418-011-0579-x |
| Ellagic acid | [5281855](https://pubchem.ncbi.nlm.nih.gov/compound/5281855) | Inhibition of violacein and AHL production | 10.1016/j.foodcont.2011.09.006 |
| Epicatechin | 72276 | Antimicrobial activity and inhibition of violacein | 10.1080/08927014.2013.852542 |
| Epigallocatechin | [72277](https://pubchem.ncbi.nlm.nih.gov/compound/72277) | Antiabetic activity | 10.1093/jn/136.10.2512 |
| Epigallocatechin (3- gallate) | 65064 | Inhibition of violacein production and antibiofilm | 10.1038/srep16158 |
| Eriodictyol | 440735 | Inhibition of pyocyanine, elastase and violacein | 10.1099/mic.0.049338-0 |
| Ferulic acid | [445858](https://pubchem.ncbi.nlm.nih.gov/compound/445858) | Inhibition of violacein and antibacterial activity | 10.1080/08927014.2013.852542 |
| Gallic acid | [370](https://pubchem.ncbi.nlm.nih.gov/compound/370#section=Top) | Antibacterial activity | 10.1016/j.supflu.2018.07.025 |
| Genistein | 5280961 | Bacteriostatic activity and inhibition of toxin production | 10.1007 / s12272-010-0520-y |
| Gentisic acid | [3469](https://pubchem.ncbi.nlm.nih.gov/compound/3469#section=Top) | Radioprotective property | 10.3109/10715762.2011.633518 |
| Gentisin | [5281636](https://pubchem.ncbi.nlm.nih.gov/compound/5281636) | Antibacterial and antioxidant activity | 10.1016/j.micpath.2017.10.049 |
| Ginkgetin | 5271805 | Anti-inflammatory, antiviral and antifungal. | 10.3109 / 10715762.2015.1032958 |
| Globuxanthone | [60148490](https://pubchem.ncbi.nlm.nih.gov/compound/60148490) | Antibacterial activity | [10.1016/j.phytol.2017.05.014](https://doi.org/10.1016/j.phytol.2017.05.014) |
| Glycitein | 5317750 | Antibacterial, antitumor and anticancer activity | PMID: 26339345; PMCID: PMC4555673 |
| Hesperidin | 10621 | Inhibition of HSL and violacein production | 10.1021/jf301365a |
| Isorhamnetin | [5281654](https://pubchem.ncbi.nlm.nih.gov/compound/5281654) | Antiviral, antimicrobial and anti-inflammatory activity | 10.1158 / 0008-5472.CAN-13-0525 |
| Kaempferol | 5280863 | Antimicrobial activity, inhibition of AI-1 and AI-2 | 10.1111/j.1365-2672.2010.04677.x |
| Lapachol | 3884 | Antiviral, antibacterial and antifungal, anti-inflammatory | 10.1016/j.bmcl.2013.12.049 |
| Luteoforol | 114505 | Antibacterial and antifungal activity | 10.1007/s10658-005-2192-x |
| Luteolin | 5280445 | Antibacterial activity, inhibition of HSL production and motility | 10.1007/s11104-015-2659-2 |
| Malvidin | 159287 | Antibiofilm activity, inhibition of violacein and EPS production | 10.1016/j.micpath.2015.01.010 |
| Malvidin-3-glucoside | [443652](https://pubchem.ncbi.nlm.nih.gov/compound/443652) | Anti-inflammatory activity | 10.1016/j.foodchem.2009.12.097 |
| Mangiferin | [5281647](https://pubchem.ncbi.nlm.nih.gov/compound/5281647) | Antidiabetic | 10.1078/0944-7113-00009 |
| Mangostin | [5281650](https://pubchem.ncbi.nlm.nih.gov/compound/5281650) | Antibacterial and antifungal activity | 10.1016/j.phymed.2003.09.012 |
| Matairesinol | 119205 | Anti-inflammatory activity | 10.1007/s11064-017-2301-1 |
| Methyl 4-hydroxycinnamate | [5319562](https://pubchem.ncbi.nlm.nih.gov/compound/5319562#section=Top) | Antibacterial activity | 10.1111/1750-3841.13302 |
| Morelloflavone | 5464454 | Anti-inflammatory, antibacterial and hypocholesterolemic action | 10.1002/ptr.3286 |
| Myricetin | 5281672 | Inhibition of pyocyanine and antibiofilm activity | 10.1016/j.micpath.2017.07.018 |
| Naringenin | 932 | Inhibition of pyocyanine, elastase, production of HSL | 10.1099/mic.0.049338-0 |
| P-coumaric acid | 637775 | Antibacterial and anti-QS activity | 10.1089/jmf.2012.0197 |
| Pelargonidin | 440832 | Antibiofilm activity | 10.1080/14786419.2016.1222386 |
| Peonidin | 441773 | Prebiotic and antibacterial activity | 10.1038/s41598-018-23397-0 |
| Peonidin-3-glucoside | [443654](https://pubchem.ncbi.nlm.nih.gov/compound/443654) | Anticancer activity | 10.1080/01635580903441261 |
| Petunidin | 441774 | Antibiofilm activity, inhibition of EPS production | 10.1039 / C5RA20677D |
| Phloretin | 4788 | Antibacterial activity | 10.1016/j.foodchem.2014.03.118 |
| Piceatannol | 667639 | Anti-inflammatory and anticancer, therapeutic potential | 10.1089/jmf.2017.3916 |
| Procyanidin | 107876 | Anti hypertensive | 10.1016/j.foodres.2013.01.023 |
| Procyanidin B1 | [11250133](https://pubchem.ncbi.nlm.nih.gov/compound/11250133) | Anti-inflammatory activity | 10.1007/s11010-015-2457-4 |
| Pyrocatechol | 289 | Chemopreventive | 10.1016 / j.fct.2019.02.010 |
| Quercetin | 5280343 | Antimicrobial activity, inhibition of AI-1 and AI-2 | 10.1111/j.1365-2672.2010.04677.x |
| Quercetin-3,4-diglucoside | [11215769](https://pubchem.ncbi.nlm.nih.gov/compound/11215769) | Antibacterial activity | 10.3389/fmicb.2019.00867 |
| Quercetin-3-glucoside | [44259229](https://pubchem.ncbi.nlm.nih.gov/compound/44259229) | Antibacterial activity | 10.4314 / tjpr.v12i5.23 |
| Quercetin-3-β-D-glucoside | [5280804](https://pubchem.ncbi.nlm.nih.gov/compound/5280804) | Anticancer activity | 10.1021/jf8039796 |
| Quercitrin | [5280459](https://pubchem.ncbi.nlm.nih.gov/compound/5280459) | Anti-inflammatory activity | 10.1038/sj.bjp.0705941 |
| Resorcinol | 5054 | Antibacterial and anticancer activity | 10.1016/j.jorganchem.2014.12.042 |
| Resveratrol | [445154](https://pubchem.ncbi.nlm.nih.gov/compound/445154#section=Top) | Inhibition of the production of violacein and HSL | 10.1016/j.foodcont.2011.09.006 |
| Rutin | 5280805 | Inhibition of pyocyanine and antibiofilm activity | 10.1016/j.micpath.2017.07.018Obtenha direitos e conteúdo |
| Salicylic acid | [338](https://pubchem.ncbi.nlm.nih.gov/compound/338) | Antimicrobial activity, inhibition of AHL, proteases and motility | 10.1128 / AAC.01283-08 |
| Sciadopitysin | 5281696 | Anti-inflammatory activity | 10.1002/jat.3620 |
| Secoisolariciresinol | [65373](https://pubchem.ncbi.nlm.nih.gov/compound/Secoisolariciresinol) | Anticancer and antidiabetic activity | 10.1016/j.biopha.2016.07.041 |
| Sinapic acid | 637775 | Antibacterial activity | 10.1016/j.foodchem.2007.07.003 |
| Syringic acid | [10742](https://pubchem.ncbi.nlm.nih.gov/compound/10742) | Antibacterial and antifungal activity | 10.5539 / jas.v1n2p15 |
| Taxifolin | 439533 | Inhibition of pyocyanin, elastase and expression of QS genes | 10.1099/mic.0.049338-0 |
| Theaflavin | 135403798 | Antibacterial activity | 10.1016/j.ijantimicag.2011.07.006 |
| Theograndin | [11156985](https://pubchem.ncbi.nlm.nih.gov/compound/11156985#section=Top) | Antioxidant activity | <https://doi.org/10.1021/np034002j> |
| Vanillic acid | [8468](https://pubchem.ncbi.nlm.nih.gov/compound/8468) | Inhibition of violacein production | 10.1016/j.foodcont.2011.09.006 |
| Vanillin | [1183](https://pubchem.ncbi.nlm.nih.gov/compound/1183#section=Top) | Antimutagenic, , anti-colitis and analgesic effect | 10.1016/j.bbagen.2010.11.004 |
| Viniferin | [5315232](https://pubchem.ncbi.nlm.nih.gov/compound/5315232#section=Top) | Anti-inflammatory and anticancer activity | 10.3390/molecules22050733 |
| Volkensiflavone | 23844069 | Antibacterial activity | 10.1016/j.fitote.2003.12.023 |

**Supplementary Table 2:** Molecular docking of compounds with better binding affinity with CviR protein structures (Part 1: 3QP1, 3QP2, 3QP4).

| **Molecule** | **Type** | **Pubchem CID** | **3QP1** | | | | | **3QP2** | | | | | **3QP4** | | | | | |
| --- | --- | --- | --- | --- | --- | --- | --- | --- | --- | --- | --- | --- | --- | --- | --- | --- | --- | --- |
|  |  |  | **Binding residue** | **Hydrogen bond score** | **Steric interaction score** | **GScore** | **Rank** | **Binding residue** | **Hydrogen bond score** | **Steric interaction score** | **GScore** | **Rank** | **Binding residue** | **Hydrogen bond score** | **Steric interaction score** | **GScore** | **Rank** |  |
| N-(3-oxododecanoyl)-L-homoserine lactone | AHL | 3246941 | Y80, W84, Y88, D97, S155 | -10,00 | -77,53 | -83,91 | 1 | Y80, W84, Y88, D97, S155 | -10,00 | -77,26 | -82,53 | 3 | Y80, W84, Y88, D97, S155 | -9,87 | -79,46 | -85,61 | 1 |  |
| N-(3-hydroxydodecanoyl)-DL-homoserine lactone | AHL | 11507677 | Y80, W84, Y88, D97, S155 | -10,00 | -78,69 | -82,29 | 2 | Y80, W84, D97, S155 | -9,49 | -78,24 | -83,73 | 1 | Y80, W84, D97, S155 | -10 | -79,94 | -84,55 | 3 |  |
| N-dodecanoyl-DL-homoserine lactone | AHL | 11565426 | Y80, W84, D97, S155 | -8,00 | -78,52 | -81,99 | 3 | Y80, W84, D97, S155 | -8,00 | -79,34 | -82,61 | 2 | Y80, W84, D97, S155 | -8 | -81,3 | -85,43 | 2 |  |
| Demethoxycurcumin | Polyphenol | 5469424 | M135 | -1,00 | -82,22 | -80,73 | 4 | M135 | -1,3 | -82,05 | -80,76 | 4 | M135 | -0,52 | -82,36 | -80,79 | 7 |  |
| Bisdemethoxycurcumin | Polyphenol | 5315472 | M135 | -1,59 | -79,65 | -79,25 | 5 | M135 | -1,73 | -78,99 | -78,92 | 5 | M135 | -0,68 | -81,26 | -80,49 | 8 |  |
| N-(3-hydroxydecanoyl)-DL-homoserine lactone | AHL | 71353010 | Y80, W84, D97, S155 | -10,00 | -72,18 | -79,00 | 6 | Y80, W84, Y88, D97, S155 | -10,00 | -73,92 | -78,08 | 7 | Y80, W84, Y88, D97, S155 | -8,8 | -76,31 | -81,61 | 4 |  |
| N-(3-oxodecanoyl)-L-homoserine lactone | AHL | 10221060 | Y80, W84, Y88, D97, S155 | -9,86 | -71,88 | -77,80 | 7 | Y80, W84, D97, S155 | -8,00 | -73,49 | -78,50 | 6 | Y80, W84, D97, S155 | -8 | -75,77 | -80,87 | 6 |  |
| N-decanoyl-DL-homoserine lactone | AHL | 11644562 | Y80, W84, D97, S155 | -8,00 | -74,05 | -77,43 | 8 | Y80, W84, D97, S155 | -8,00 | -73,11 | -77,77 | 8 | Y80, W84, D97, S155 | -8 | -75,71 | -81,4 | 5 |  |
| N-(3-oxooctanoyl)-L-homoserine lactone | AHL | 127293 | Y80, W84, Y88, D97, S155 | -10,00 | -67,36 | -74,29 | 9 | Y80, W84, Y88, D97, S155 | -10,00 | -66,65 | -73,09 | 11 | Y80, W84, Y88, D97, S155 | -10 | -67,2 | -73,81 | 12 |  |
| 4-bromo-5-(bromomethylene)-3-dodecyl-2(5H)-furanone | Antagonist | 10180544 | W84 | -4,00 | -73,35 | -73,68 | 10 | S155 | -2 | -74,01 | -73,76 | 10 | S155 | -2 | -76,83 | -77,4 | 10 |  |
| N-octanoyl-DL-homoserine lactone | AHL | 3474204 | Y80, W84, D97, S155 | -8,00 | -66,75 | -73,26 | 11 | Y80, W84, S155 | -8,00 | -66,11 | -72,47 | 12 | Y80, S155 | -8 | -66,43 | -73,19 | 13 |  |
| Dihydrocapsaicin | Biflavonoid | 107982 | Y88 | -4,00 | -72,21 | -71,64 | 12 | W84, Y88 | -6,00 | -70,39 | -72,38 | 13 | Y80, W84, S155 | -5,74 | -70,41 | -72,62 | 15 |  |
| Matairesinol | Lignan | 119205 | W84, Y88, D97 | -5,11 | -69,79 | -70,49 | 13 | W84,Y88, D97 | -5,35 | -74,63 | -76,76 | 9 | W84, Y88, D97 | -4,48 | -76,65 | -77,9 | 9 |  |
| **Capsaicin** | **Polyphenol** | **1548943** | **W84, Y88** | **-4,00** | **-71,98** | **-70,39** | **14** | **Y88, N92** | **-1,71** | **-73,06** | **-70,65** | **15** | **W84, Y88** | **-4** | **-72,4** | **-71,74** | **16** |  |
| **Curcumin** | **Polyphenol** | **969516** | **N92** | **-4,00** | **-66,89** | **-66,93** | **17** | **W84** | **-2,18** | **-74,33** | **-71,54** | **14** | **N92** | **-0,83** | **-76,01** | **-73,12** | **14** |  |
| N-(3-hydroxyhexanoyl)-DL-homoserine lactone | AHL | 70185030 | Y80, W84, Y88, D97, S155 | -9,84 | -58,56 | -66,60 | 18 | Y80, W84, Y88, D97, S155 | -10,00 | -57,65 | -65,71 | 24 | Y80, W84, Y88, S155 | -9,63 | -58,67 | -66,49 | 28 |  |
| N-(3-oxohexanoyl)-L-homoserine lactone | AHL | 688505 | Y80, W84, Y88, D97, S155 | -10,00 | -59,19 | -66,57 | 19 | Y80, W84, Y88, D97, S155 | -9,43 | -57,93 | -64,58 | 31 | Y80, W84, Y88, D97, S155 | -9,73 | -59,1 | -66,22 | 31 |  |
| N-(3-hydroxyoctanoyl)-DL-homoserine lactone | AHL | 11586792 | W84, Y88, D97 | -9,80 | -59,10 | -65,65 | 21 | Y80, Y88, S155 | -7,72 | -60,94 | -66,46 | 22 | Y80, Y88, S155 | -8 | -60,52 | -66,47 | 29 |  |
| Secoisolariciresinol | Lignan | 65373 | W84, D97 | -3,16 | -66,44 | -64,93 | 23 | W84 | -2 | -73,98 | -70,05 | 16 | Y88, D97 | -4 | -73,91 | -69,96 | 18 |  |
| N-hexanoyl-DL-homoserine lactone | AHL | 3462373 | Y80, W84, D97, S155 | -8,00 | -58,09 | -64,90 | 24 | Y80, W84, D97, S155 | -8,00 | -57,33 | -63,91 | 33 | Y80, W84, D97, S155 | -8 | -57,94 | -64,77 | 32 |  |
| **Phloretin** | **Polyphenol** | **4788** | **Y88, M135** | **-3,81** | **-67,71** | **-64,84** | **25** | **M135** | **-2** | **-69,74** | **-65,66** | **25** | **Y88, M135** | **-4** | **-68,96** | **-66,4** | **30** |  |
| Mangostin | Xantone | 5281650 | Y80, S155 | -3,84 | -57,02 | -57,70 | 37 | Y80, S155 | -4 | -66,88 | -67,34 | 18 | Y80, S155 | -3,94 | -67,28 | -67,79 | 23 |  |
| Naringenin | Flavonoid | 42607905 | L85 | -2,00 | -45,48 | -43,34 | 61 | Y80, Y88, D97, S155 | -8,61 | -53,93 | -60,85 | 39 | L85, M135 | -1,27 | -67,77 | -67,84 | 22 |  |

Selected phenolic compounds are in bold.

**Supplementary Table 3:** Molecular docking of compounds with better binding affinity with CviR protein structures (Part 2: 3QP5, 3QP6, 3QP8).

| **Molecule** | **Type** | **Pubchem CID** | **3QP5** | | | | | **3QP6** | | | | | **3QP8** | | | | |
| --- | --- | --- | --- | --- | --- | --- | --- | --- | --- | --- | --- | --- | --- | --- | --- | --- | --- |
|  |  |  | **Binding residue** | **Hydrogen bond score** | **Steric interaction score** | **GScore** | **Rank** | **Binding residue** | **Hydrogen bond score** | **Steric interaction score** | **GScore** | **Rank** | **Binding residue** | **Hydrogen bond score** | **Steric interaction score** | **GScore** | **Rank** |
| N-(3-oxododecanoyl)-L-homoserine lactone | AHL | 3246941 | W84, D97, S155 | -5,99 | -73,9 | -75,2 | 7 | Y80,W84, Y88, D97, S155 | -10 | -83,75 | -89,6 | 1 | Y80, W84, D97, S155 | -8 | -81,38 | -85,6 | 3 |
| N-(3-hydroxydodecanoyl)-DL-homoserine lactone | AHL | 11507677 | W84, Y88, D97, S155 | -10 | -70,01 | -75,4 | 6 | Y80, W84, Y88, D97, S155 | -10 | -83,25 | -88,97 | 2 | Y80, W84, S155 | -10 | -79,99 | -86,2 | 2 |
| N-dodecanoyl-DL-homoserine lactone | AHL | 11565426 | W84, D97, S155 | -6 | -71,8 | -72,03 | 11 | Y80, W84, D97, S155 | -8 | -82,31 | -87,18 | 3 | Y80, W84, S155 | -7,53 | -81,67 | -86,87 | 1 |
| Demethoxycurcumin | Polyphenol | 5469424 | L85, M135 | -3,41 | -69,13 | -71,17 | 15 | S89, M135 | -4 | -87,95 | -85,33 | 4 | S89, M135 | -7,62 | -77,15 | -81,58 | 5 |
| Bisdemethoxycurcumin | Polyphenol | 5315472 | L85, M135 | -3,53 | -68,36 | -71,33 | 14 | Y88, M135 | -3,27 | -83,58 | -83,36 | 7 | Y80, M135, S155 | -6 | -78,12 | -81,99 | 4 |
| N-(3-hydroxydecanoyl)-DL-homoserine lactone | AHL | 71353010 | W84, Y88, D97, S155 | -8,63 | -63,32 | -68,93 | 21 | Y80, W84, Y88, D97, S155 | -10 | -77,17 | -85,11 | 5 | Y80, W84, Y88, D97, S155 | -9,85 | -74,01 | -81,54 | 6 |
| N-(3-oxodecanoyl)-L-homoserine lactone | AHL | 10221060 | W84, D97, S155 | -5,96 | -67,54 | -70,1 | 18 | Y80, W84, Y88, D97, S155 | -10 | -77,23 | -83,99 | 6 | Y80, W84, Y88, D97, S155 | -9,96 | -74,07 | -80,88 | 8 |
| N-decanoyl-DL-homoserine lactone | AHL | 11644562 | W84, D97, S155 | -6 | -66,79 | -69,89 | 19 | Y80, W84, D97, S155 | -8 | -77 | -82,21 | 8 | Y80, W84, S155 | -8 | -75,61 | -81,28 | 7 |
| N-(3-oxooctanoyl)-L-homoserine lactone | AHL | 127293 | W84, Y88, D97, S155 | -8 | -60,09 | -65,42 | 25 | Y80, W84, Y88, D97, S155 | -10 | -68,58 | -75,47 | 11 | Y80, W84, Y88, D97, S155 | -9,96 | -66,93 | -74,21 | 12 |
| 4-bromo-5-(bromomethylene)-3-dodecyl-2(5H)-furanone | Antagonist | 10180544 | - | 0 | -80,21 | -75,52 | 5 | S155 | -2 | -77,57 | -76,86 | 9 | S155 | -2 | -74,37 | -72,78 | 17 |
| N-octanoyl-DL-homoserine lactone | AHL | 3474204 | W84, D97, S155 | -6 | -59,8 | -63,52 | 27 | Y80, W84, S155 | -8 | -68,38 | -75,11 | 13 | Y80, W84, S155 | -7,77 | -68,25 | -73,55 | 15 |
| Dihydrocapsaicin | Polyphenol | 107982 | Y88 | 0 | -74,53 | -70,5 | 17 | S89 | -1,95 | -76,76 | -75,42 | 12 | S89 | -0,75 | -72,46 | -69,51 | 16 |
| Matairesinol | Lignan | 119205 | N92, M89 | -3,08 | -77,44 | -77,27 | 3 | W84, Y88, D97 | -5,02 | -70,45 | -72,2 | 15 | W84, Y88, D97 | -5,56 | -71,52 | -73,76 | 14 |
| **Capsaicin** | **Polyphenol** | **1548943** | **W84** | **-2** | **-73,36** | **-70,62** | **16** | **S89** | **-1,89** | **-77,38** | **-76,67** | **10** | **S89** | **-1,68** | **-76,08** | **-73,8** | **13** |
| **Curcumin** | **Polyphenol** | **969516** | **N92** | **-4** | **-72,51** | **-73,05** | **8** | **N92** | **-2** | **-78,26** | **-73,37** | **14** | **W84** | **-3,54** | **-77,12** | **-75,82** | **11** |
| N-(3-hydroxyhexanoyl)-DL-homoserine lactone | AHL | 70185030 | W84, S155 | -6 | -54,28 | -58,11 | 43 | Y80, W84, Y88, D97, S155 | -9,95 | -60,56 | -69,15 | 18 | Y80, W84, Y88, S155 | -9,96 | -60,42 | -69,01 | 23 |
| N-(3-oxohexanoyl)-L-homoserine lactone | AHL | 688505 | W84, Y88, D97, S155 | -7,98 | -53,55 | -58,97 | 40 | Y80, W84, Y88, D97, S155 | -10 | -60,08 | -67,4 | 20 | Y80, W84, Y88, D97, S155 | -10 | -59,6 | -66,67 | 25 |
| N-(3-hydroxyoctanoyl)-DL-homoserine lactone | AHL | 11586792 | Y80, Y88, D97 | -5,32 | -57,67 | -60,9 | 34 | Y88, Y80, S155 | -7,73 | -63,05 | -68,87 | 19 | Y80, Y88, S155 | -7,18 | -64,22 | -69,18 | 22 |
| Secoisolariciresinol | Lignan | 65373 | Y80, W84, S155 | -7,08 | -61,61 | -65,55 | 24 | S89 | -3,78 | -76,39 | -70,2 | 16 | D97 | -2 | -70,84 | -69,9 | 21 |
| N-hexanoyl-DL-homoserine lactone | AHL | 3462373 | W84, S155 | -5,87 | -53,6 | -57,53 | 44 | W84, Y80, D97, S155 | -8 | -60,47 | -66,92 | 21 | Y80, W84, S155 | -7,87 | -60,43 | -66,69 | 24 |
| **Phloretin** | **Flavonoid** | **4788** | **W84, Y88, D98, M135** | **-8** | **-62,54** | **-63,1** | **28** | **Y80, W84, M135, S155** | **-7,59** | **-70,51** | **-70,19** | **17** | **M135** | **-1,36** | **-70,07** | **-65,29** | **27** |
| Mangostin | Xantone | 5281650 | W84,Y88 | -2,44 | -82,17 | -79,29 | 1 | Y80, S155 | -5,51 | -63,46 | -64,39 | 23 | Y80, S155 | -4 | -65,14 | -65,72 | 26 |
| Naringenin | Flavonoid | 42607905 | Y80, Y88, D97, S155 | -12,19 | -61,51 | -71,69 | 12 | L85, S89, M135, S155 | -9,77 | -51,62 | -59,65 | 34 | M135 | -0,68 | -67 | -64,89 | 28 |

Selected phenolic compounds are in bold.

**SUPPLEMENTARY FIGURES**


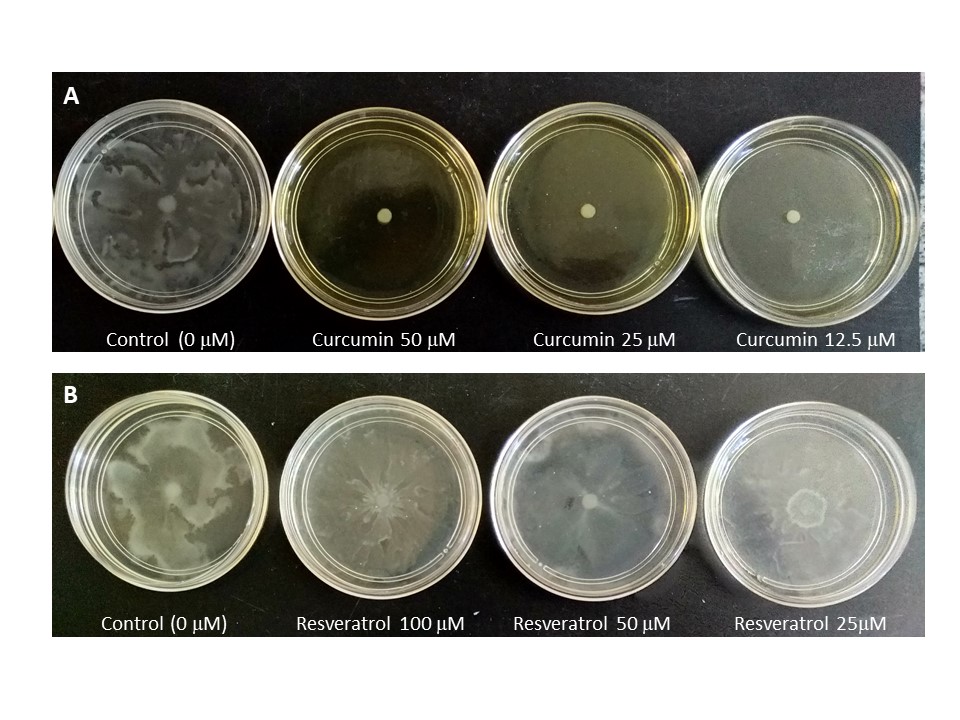


**Figure 1S**: Swarming motility of *Serratia marcescens* MG1 in semi-solid M8 agar 0.5% (w/v) with curcumin (A) and resveratrol (B).


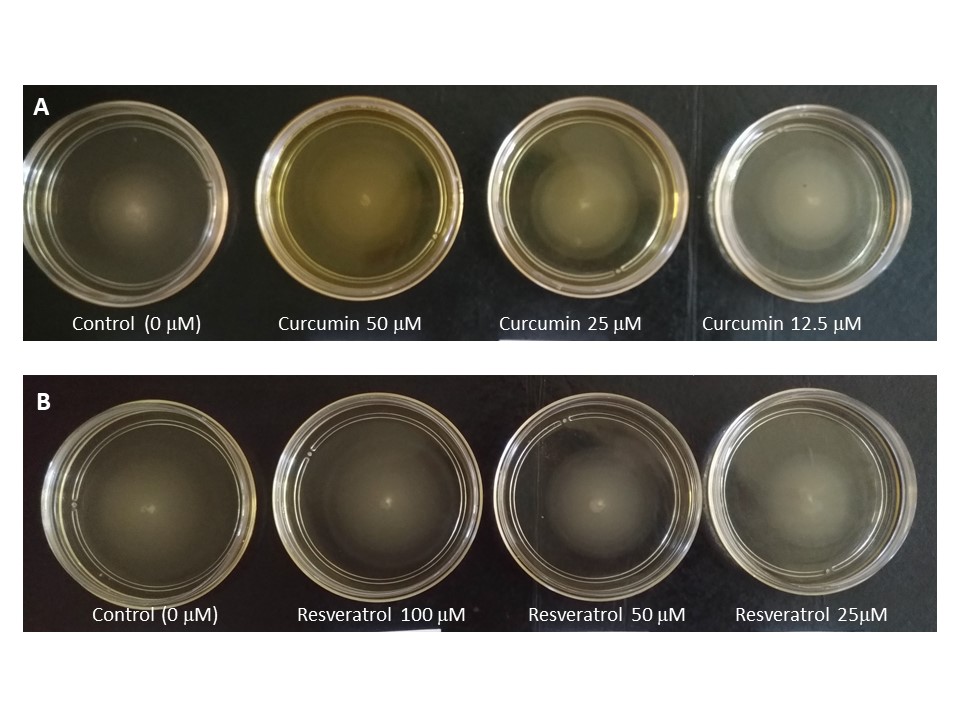


**Figure 2S**: Swimming motility of *Serratia marcescens* MG1 in semi-solid M8 agar 0.3% (w/v) with curcumin (A) and resveratrol (B).

**
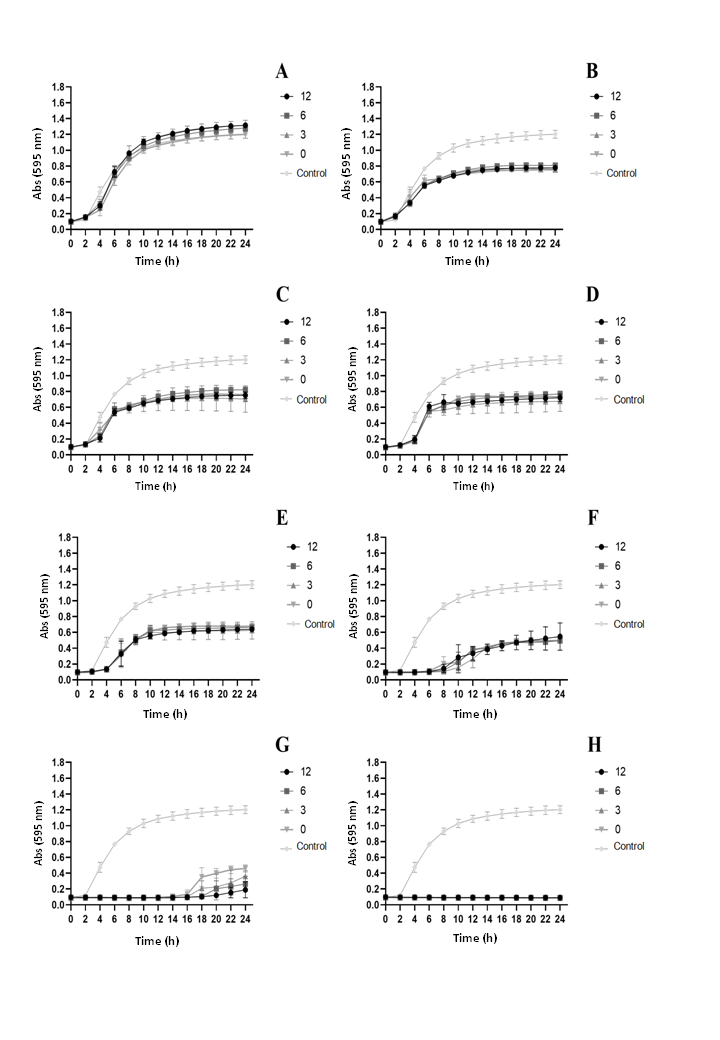
**

**Figure 3S:** Growth curves for *A. hydrophila* IOC / FDA 110-36 during 24h incubation at 37 °C in different concentrations of **curcumin** (0, 3, 6 and 12 μM) and kanamycin antibiotic indicated by the letters: (A) 4 μg/ml; (B) 8 μg ml; (C) 16 μg/ml; (D) 32 μg/ml; (E) 64 μg/ ml; (F) 128 μg/ml; (G) 256 μg/ml; (H) 512 μg/ml. Control = bacterial growth in LB with DMSO at 1% which was used to dilute the compounds.

**
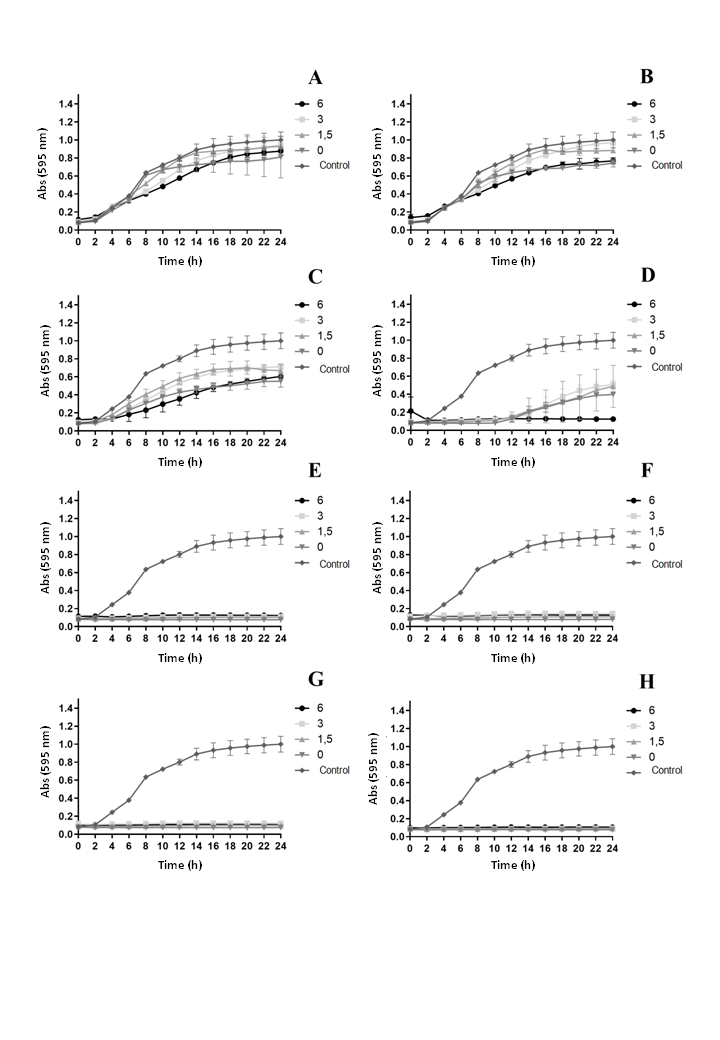
**

**Figure 4S:** Growth curves for *S.* Montevideo during 24h incubation at 37 °C in different concentrations of **curcumin** (0, 1.5, 3 and 6 μM) and kanamycin antibiotic indicated by the letters: (A) 4 μg/ml; (B) 8 μg ml; (C) 16 μg/ml; (D) 32 μg/ml; (E) 64 μg/ ml; (F) 128 μg/ml; (G) 256 μg/ml; (H) 512 μg/ml. Control = bacterial growth in LB with DMSO at 1% which was used to dilute the compounds.

**
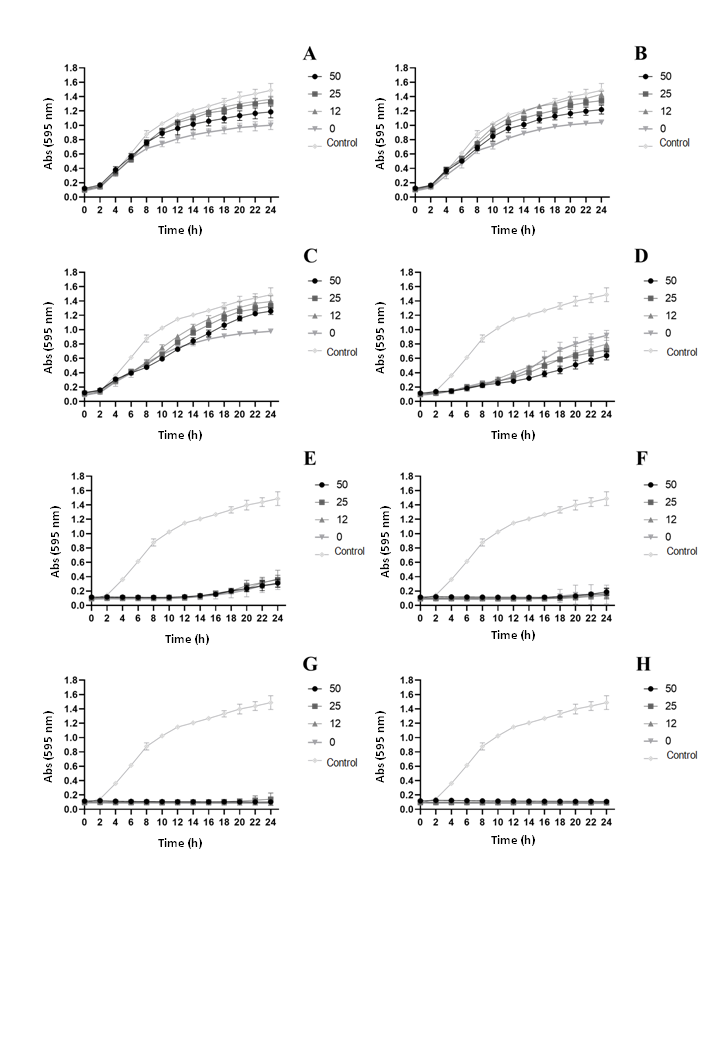
**

**Figure 5S:** Growth curves for *S. marcescens* during 24h incubation at 30 °C in different concentrations of **curcumin** (50, 25 and 12 μM) and kanamycin antibiotic indicated by the letters: (A) 4 μg/ml; (B) 8 μg ml; (C) 16 μg/ml; (D) 32 μg/ml; (E) 64 μg/ ml; (F) 128 μg/ml; (G) 256 μg/ml; (H) 512 μg/ml. Control = bacterial growth in LB with DMSO at 1% which was used to dilute the compounds.

**
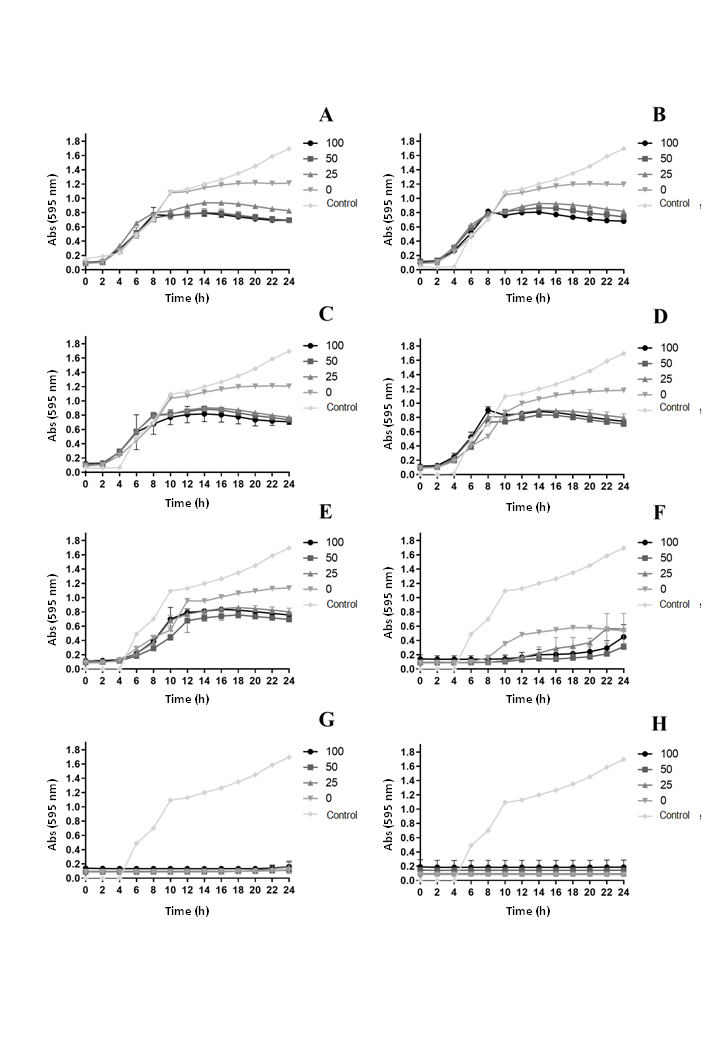
**

**Figure 6S:** Growth curves for *A. hydrophila* during 24h incubation at 37 °C in different concentrations of **resveratrol** (100, 50 and 25 μM) and kanamycin antibiotic indicated by the letters: (A) 4 μg/ml; (B) 8 μg ml; (C) 16 μg/ml; (D) 32 μg/ml; (E) 64 μg/ ml; (F) 128 μg/ml; (G) 256 μg/ml; (H) 512 μg/ml. Control = bacterial growth in LB with DMSO at 1% which was used to dilute the compounds.

**
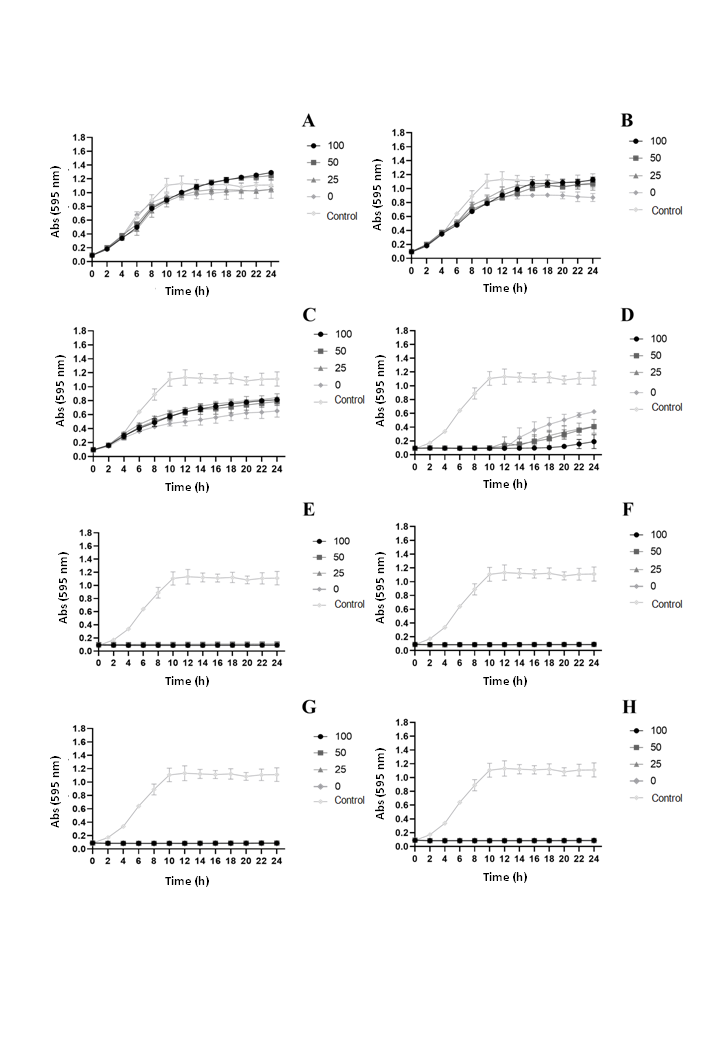
 Figure 7S:** Growth curves for *S.* Montevideo during 24h incubation at 37 °C in different concentrations of **resveratrol** (100, 50 and 25 μM) and kanamycin antibiotic indicated by the letters: (A) 4 μg/ml; (B) 8 μg ml; (C) 16 μg/ml; (D) 32 μg/ml; (E) 64 μg/ ml; (F) 128 μg/ml; (G) 256 μg/ml; (H) 512 μg/ml. Control = bacterial growth in LB with DMSO at 1% which was used to dilute the compounds.

**
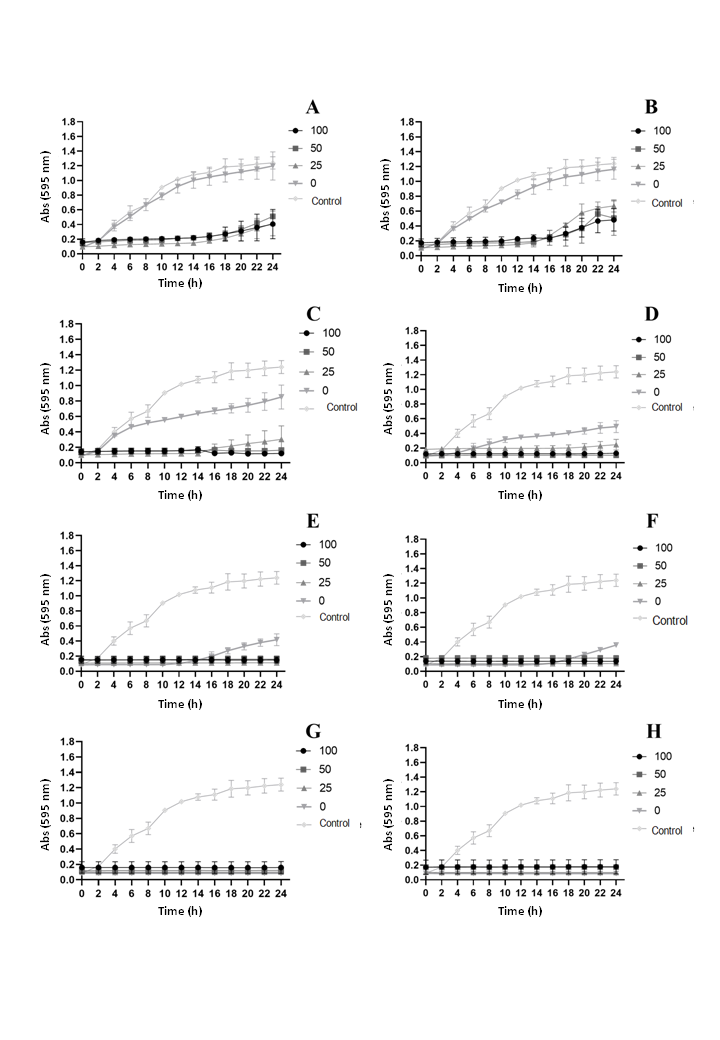
 Figure 8S:** Growth curves for *S. marcescens* during 24h incubation at 30 °C in different concentrations of **resveratrol** (100, 50 and 25 μM) and kanamycin antibiotic indicated by the letters: (A) 4 μg/ml; (B) 8 μg ml; (C) 16 μg/ml; (D) 32 μg/ml; (E) 64 μg/ ml; (F) 128 μg/ml; (G) 256 μg/ml; (H) 512 μg/ml. Control = bacterial growth in LB with DMSO at 1% which was used to dilute the compounds.
